# Chemosensory sensilla of the *Drosophila* wing express a candidate ionotropic pheromone receptor

**DOI:** 10.1371/journal.pbio.2006619

**Published:** 2019-05-21

**Authors:** Zhe He, Yichen Luo, Xueying Shang, Jennifer S. Sun, John R. Carlson

**Affiliations:** Department of Molecular, Cellular, and Developmental Biology, Yale University, New Haven, Connecticut, United States of America; Brandeis University, United States of America

## Abstract

The *Drosophila* wing was proposed to be a taste organ more than 35 years ago, but there has been remarkably little study of its role in chemoreception. We carry out a differential RNA-seq analysis of a row of sensilla on the anterior wing margin and find expression of many genes associated with pheromone and chemical perception. To ask whether these sensilla might receive pheromonal input, we devised a dye-transfer paradigm and found that large, hydrophobic molecules comparable to pheromones can be transferred from one fly to the wing margin of another. One gene, *Ionotropic receptor* (*IR*)*52a*, is coexpressed in neurons of these sensilla with *fruitless*, a marker of sexual circuitry; *IR52a* is also expressed in legs. Mutation of *IR52a* and optogenetic silencing of IR52a^+^ neurons decrease levels of male sexual behavior. Optogenetic activation of IR52a^+^ neurons induces males to show courtship toward other males and, remarkably, toward females of another species. Surprisingly, *IR52a* is also required in females for normal sexual behavior. Optogenetic activation of IR52a^+^ neurons in mated females induces copulation, which normally occurs at very low levels. Unlike other chemoreceptors that act in males to inhibit male–male interactions and promote male–female interactions, IR52a acts in both males and females, and can promote male–male as well as male–female interactions. Moreover, IR52a^+^ neurons can override the circuitry that normally suppresses sexual behavior toward unproductive targets. Circuit mapping and Ca^2+^ imaging using the *trans*-Tango system reveals second-order projections of IR52a^+^ neurons in the subesophageal zone (SEZ), some of which are sexually dimorphic. Optogenetic activation of IR52a^+^ neurons in the wing activates second-order projections in the SEZ. Taken together, this study provides a molecular description of the chemosensory sensilla of a greatly understudied taste organ and defines a gene that regulates the sexual circuitry of the fly.

## Introduction

Taste, or contact chemoreception, allows insects to detect an immense variety of environmental cues [1]. These cues may signify the presence of nutrients, toxins, competitors, or mates [2]. A better understanding of insect chemoreception could lead to new means of controlling agricultural pests and disease vectors that inflict enormous global damage [3].

*Drosophila melanogaster* is an excellent model for the study of insect contact chemoreception. Taste organs of the fly contain sensilla that have a pore at the tip [4]. When a sensillum makes contact with the environment, chemical compounds diffuse through the pore and activate gustatory receptor (Gr) neurons within. Each sensillum is innervated by several gustatory neurons and often one mechanosensory neuron. Different taste neurons respond to different stimuli, such as sugars, bitter compounds, salts, water, acids, lipids, or pheromones [5].

There have been extensive studies of several *Drosophila* taste organs: the labellum (the major taste organ of the head), the legs, and the pharynx [1]. By contrast, the wing has been believed for more than 35 years to detect tastants but has received remarkably little attention as a taste organ [6]. The wing contains a row of approximately 40 curved sensilla on the anterior margin of each face of the wing. There is evidence to support a chemosensory function for these sensilla, but the data are limited [7–9].

Within the taste organs of the fly, several classes of receptors detect chemical stimuli. The *D*. *melanogaster* genome contains 60 *Gr* genes, which encode receptors that detect bitter compounds, sugars, and other classes of stimuli [10, 11]. A family of 60 *Ionotropic receptors* (*IR*s) includes a large clade expressed in taste organs [12–21]. Among this clade are *IR52c* and *IR52d*, which are coexpressed in neurons of the male foreleg and are required for normal sexual behavior [15]; another *IR*, *IR60b*, is expressed in the pharynx and responds to sucrose [18]. Several members of the *pickpocket* (*ppk*) family are expressed in taste organs and have been implicated in pheromone recognition [1, 22–27]. Certain transient receptor potential (Trp) receptors are also sensitive to tastants [28–31].

One of the most fundamental problems in biology is how an animal identifies a suitable mating partner. Although a number of candidate pheromone receptors have been described in *Drosophila*, the number of candidate pheromones is much higher. The cuticular surface of the fly contains on the order of 50 hydrophobic hydrocarbons, some of which are transferred between male and female flies [32–34]. A major goal in the field is to identify and characterize the receptors that control the operation of sexual circuits.

Here, we use a molecular approach to identify genes expressed in chemosensory sensilla of the wing. We carry out a differential RNA-seq analysis of wild-type wings and of mutant wings that lack chemosensory sensilla. The screen identifies a large number of chemosensory genes, including genes implicated in pheromone response. To test the possibility that pheromones from one fly are transmitted to the wing margin of another fly, we carry out dye transfer experiments and find transmission to the wing. Among the genes identified in the screen is *IR52a*. Genetic analysis reveals that it is required for male sexual behavior. Optogenetic silencing and activation of IR52a^+^ neurons show that they activate a circuit that drives male sexual behavior. Strikingly, activation of these neurons drives male sexual behavior not only toward *D*. *melanogaster* females but also toward males and toward females of another species. Finally, we find that *IR52a* is highly unusual in that it is required for normal female sexual behavior as well as male behavior.

## Results

### A differential RNA-seq analysis of chemosensory sensilla of the wing

**Fig 1:**
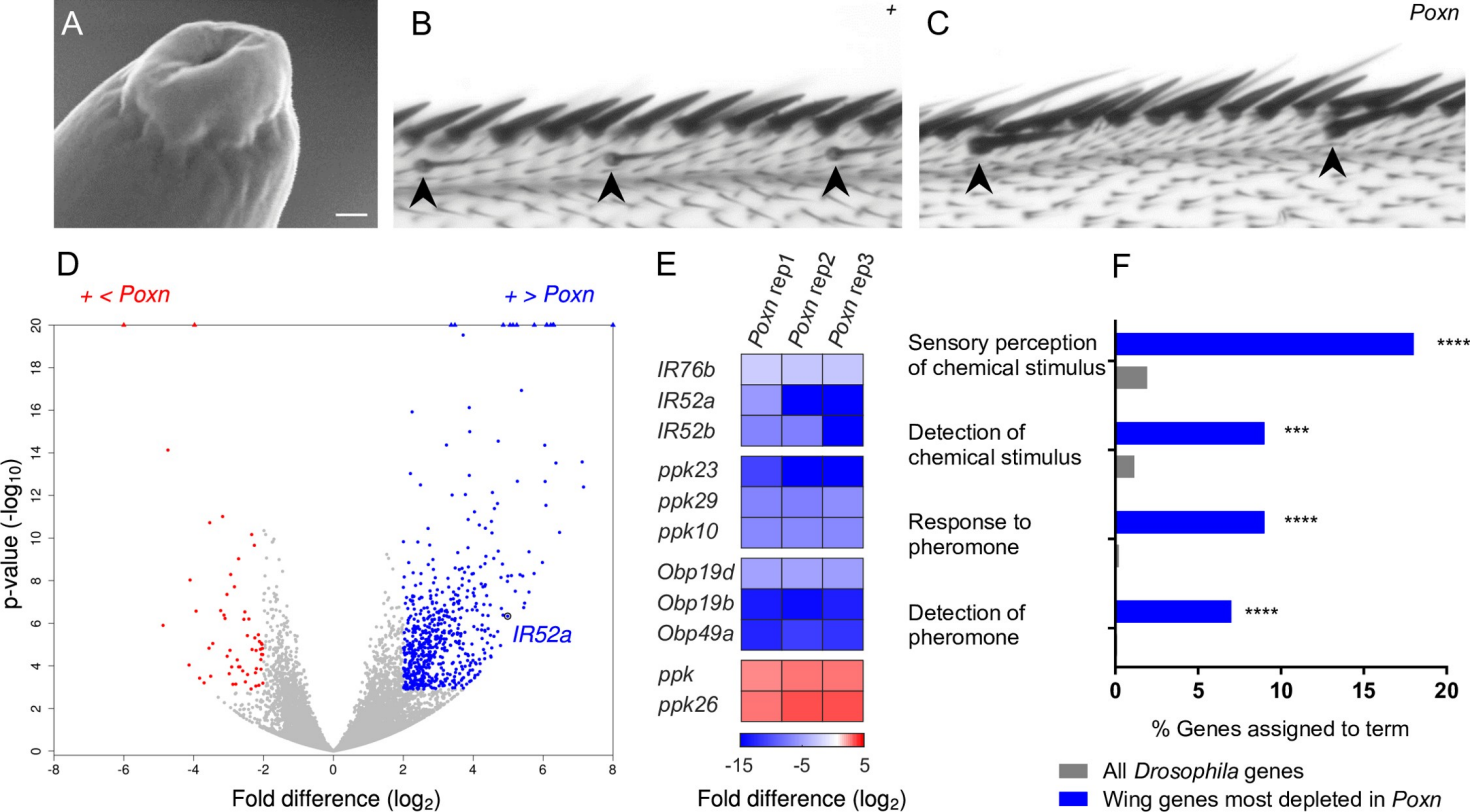
Taste sensilla in the wing and RNA-seq screen of control and *Poxn* wings. (A) Scanning electron microscope image of the tip of a chemosensory hair on the wing. Scale bar = 50 nm. (B) Anterior wing margin of a control wing (*w* Canton-S). Thin, curved chemosensory sensilla are indicated by arrowheads. (C) Anterior wing margin of *Poxn*. Chemosensory sensilla are missing; rather, sensilla that appear larger than their neighbors are observed (arrowheads). The larger sensilla may be mechanosensory. (D) Volcano plot of genes showing their relative transcript levels in *Poxn* and control wings. The x-axis indicates the log_2_ fold difference of FPKM for each gene. The y-axis indicates the *P* value of the difference. Blue dots indicate genes that are expressed at levels more than 4-fold higher in control than in Poxn and for which the difference is significant at *P* < 0.01 (adjusted *P* < 0.01, DESeq2 Wald test). Red dots indicate genes that are expressed at levels more than 4-fold higher in *Poxn*. Points at top of graph represent genes for which *P* values are extremely small. (E) Genes from the *IR*, *Ppk*, and *Obp* genes that show the most significant enrichment or depletion within their respective families. Color indicates log_2_ fold difference. (F) Gene ontology enrichment analysis of the top 100 genes that had more than a 4-fold increase in control as determined by *P* value. ***FDR < 0.001; ****FDR < 0.0001, PANTHER Overrepresentation Test. FDR, false discovery rate; FPKM, fragments per kilobase of transcript per million mapped reads; IR, ionotropic receptor.

We first examined the curved sensilla of the wing by electron microscopy to confirm that they contained the cardinal feature of a taste sensillum: a terminal pore through which tastants can enter. We examined a total of approximately 30 sensilla on three wings and observed pores (Fig 1A) comparable to those on other taste organs.

To investigate the molecular organization of the chemosensory sensilla, we carried out a differential RNA-seq analysis. We hand-dissected a total of 24,000 wings, half from control flies and half from *Poxn* mutants that lack chemosensory sensilla on the wing (Fig 1B and 1C) [35]. We performed RNA-seq on three biological replicates from each genotype.

Applying a stringent criterion for differential expression (>4x enrichment and adjusted *P* < 0.01, DESeq2 Wald Test [36]), 650 transcripts were enriched in control wings, as expected for genes preferentially expressed in chemosensory sensilla (blue dots in Fig 1D). These transcripts include members of the *IR* and *ppk* receptor families (Fig 1E, S1 and S2 Figs, S1 Table). Members of the odorant binding protein (Obp) and chemosensory protein (CSP) family were also enriched (Fig 1E and S2 Fig); some members of the Obp and CSP families are expressed in taste sensilla of the labellum and leg in addition to olfactory sensilla on the antenna [37–41]. The three *IRs*, *ppks*, and *Obps* that show the most statistically significant enrichments within each family are shown in Fig 1E. Conspicuously absent from the roster of genes enriched in wild-type wings were members of the *Gr* family (S1 Fig).

We carried out a gene ontology (GO) analysis of the 100 genes that are most significantly enriched in control wings according to p values. A number of these genes are annotated with the terms “sensory perception of chemical stimulus,” “detection of a chemical stimulus,” “response to pheromone,” and “detection of pheromone” (Fig 1F). These annotations occurred more frequently among the 100 most enriched wing genes than among the set of all *Drosophila* genes in Flybase with GO annotations (*P* < 0.001 in each of the four comparisons). These results are consistent with a chemosensory function for the wing sensilla.

We note the identification of 58 genes that, reciprocally, are enriched in the *Poxn* wing relative to control (Fig 1D, red dots; Fig 1E). There is evidence that in *Poxn* the chemosensory sensilla are transformed into mechanosensory sensilla [35, 42]. Consistent with such a transformation, the genes enriched in the *Poxn* wing include *ppk*, *ppk26*, and the Trp channel gene *painless*, all of which have been implicated in mechanoreception [43–45]. An interesting corollary of these findings is that some of the remaining 55 genes in this set could play previously unknown roles in mechanoreception.

### *IR52a* is expressed in the wing

**Fig 2:**
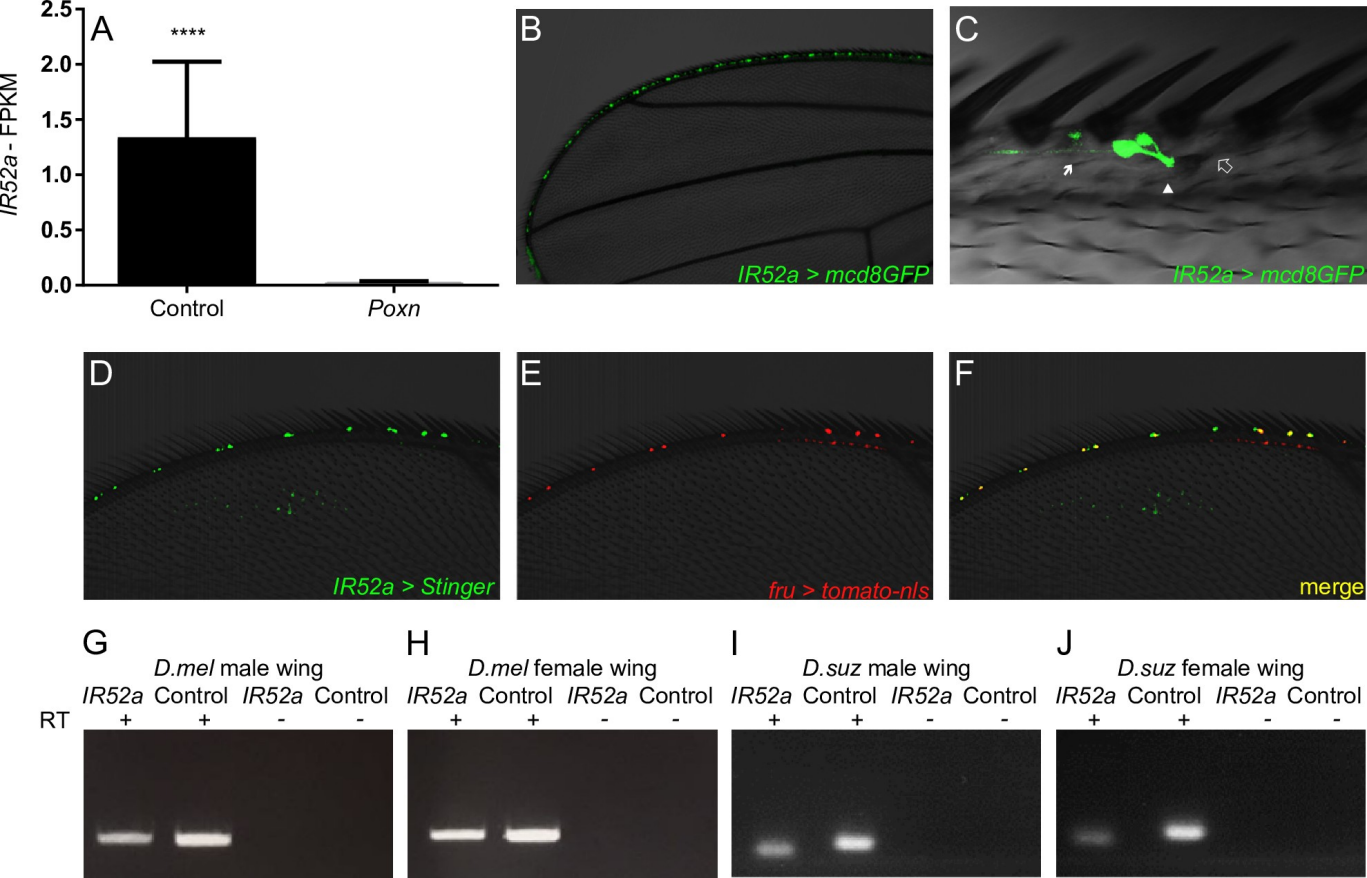
*IR52a* is expressed in *fru*^*+*^ neurons of taste sensilla in the wing. (A) FPKM of *IR52a* in control and *Poxn*. Error bars indicate SEM. ****P < 0.0001. (B) *IR52a-GAL4; UAS-mCD8-GFP*, showing expression in wing sensilla. mCD8-GFP is targeted to membranes. (C) *IR52a-GAL4* labels neurons whose dendrites (arrowhead) project into the base of a chemosensory sensillum (empty double arrow) on the wing. Arrow, axons. Two neurons innervate the indicated sensillum; some sensilla are innervated by a single labeled neuron. (D–F) Male wing labeled in green by *IR52a-GAL4*/*UAS-Stinger* (a nuclear GFP), and in red by *fru-LexA*/*LexAop-tomato-nls*. (G–J) RT-PCR of *IR52a* and *eIF1α*, used as a positive control, in wings of *D*. *melanogaster* males (G) and females (H), and in wings of *D*. *suzukii* males (I) and females (J). FPKM, fragments per kilobase of transcript per million mapped reads; IR, ionotropic receptor; RT-PCR, reverse transcriptase PCR.

Among the genes expressed at higher levels in the control wing than the *Poxn* wing was *IR52a* (Fig 2A). We confirmed the expression of *IR52a* in the wild-type wing through three additional means. First, reverse transcriptase PCR (RT-PCR) experiments detected *IR52a* in the wings of both males and females (Fig 2G and 2H). Second, quantitative PCR (qPCR) analysis with two primer pairs revealed *IR52a* expression in both male and female wings, with levels in males somewhat higher (S2 Fig; ratio = 1.3; *P* = 0.001, two-way ANOVA). Third, we confirmed and extended a previous finding that an *IR52a-GAL4* construct is expressed in cells of the anterior wing margin (Fig 2B), with similar patterns in males and females (S4A and S4B Fig) [15]. More detailed analysis revealed that *IR52a-GAL4* labels neurons that send dendrites into the chemosensory sensilla (Fig 2C). *IR52a* neurons send axons to the thoracic ganglia, where projections are visible in the wing neuropil; projections are also visible from sensilla on each of the three legs (S4C and S4D Fig)[15]. The wing neuropils of male and female appear similar, with no obvious commissure.

As a first step in investigating the functional significance of *IR52a* expression in the wing, we asked whether it is evolutionarily conserved. We carried out RT-PCR using RNA from the wing of *Drosophila suzukii* and found expression in wings of both males and females (Fig 2I and 2J). These results suggest that *IR52a* has been expressed in *Drosophila* wings for more than 25 million years.

The Fruitless (Fru) transcription factor specifies much of the circuitry that drives male sexual behavior [46–48]. Most of the wing neurons that express *IR52a-GAL4* were also observed to express *fru* (Fig 2D–2F, S5 Fig). This coexpression suggests the intriguing possibility that chemosensory sensilla in the wing play a role in sexual behavior.

### The wing receives hydrophobic compounds from another fly

**Fig 3:**
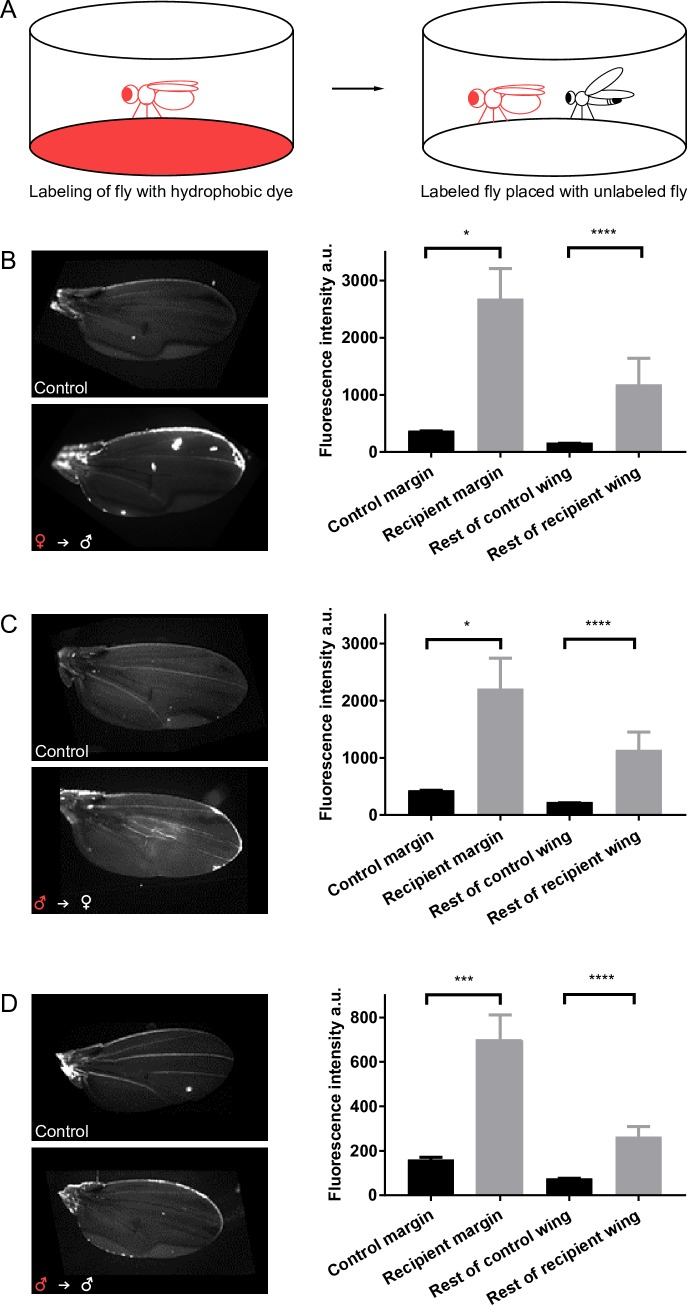
The wing receives compounds from another fly. (A) Diagram of dye transfer experiments. For simplicity, a single labeled fly is shown; in the experiments, five flies were labeled together, and five flies were placed with a single unlabeled fly. (B–D) Left: wings from a control fly and from a fly that has been in a chamber with a labeled fly. Right: fluorescence intensity in control and recipient flies. (B) Unlabeled males after being placed with labeled females, *n* = 17. (C) Unlabeled females after being placed with labeled males, *n* = 20. (D) Unlabeled males after being placed with labeled males. The y-axis in this panel is on a different scale from the other panels, *n* = 14–16. *P < 0.05; ***P < 0.001; ****P < 0.0001, Kruskal–Wallis test, Dunn's multiple comparisons test. Underlying data for this figure can be found in S1 Data.

Could large hydrophobic fly pheromones of limited volatility be transferred from one fly to the wing margin of another fly? To test this possibility, we used a large hydrophobic fluorescent compound that can be easily visualized: Nile Red (molecular weight (mw) 318 Da; for comparison, the pheromone 7-tricosene has an mw of 323 Da). We placed this dye on filter paper at the bottom of a chamber and placed flies in the chamber for 30 min, during which time they became labeled (Fig 3A and S6 Fig). We then placed labeled flies in a clean chamber with an unlabeled fly and asked whether any of the fluorescently labeled compound was transferred to the wing of the unlabeled fly (Fig 3A).

Initially, we labeled females and placed them in a chamber with unlabeled males. After 30 min, the male wing became labeled (Fig 3B). The anterior margin of the recipient males had a mean fluorescence intensity that was greater than that of control males that were placed in a chamber without labeled females. While it is difficult to quantitate the degree of labeling precisely, it is clear that the level of fluorescence in recipient males exceeded the level of autofluorescence in control males. Labeling of the recipient wing was not restricted to the anterior wing margin: we observed variable labeling, often in patches, on the rest of the wing, including the wing veins. When we measured the average fluorescence intensity of the rest of the wing, it was again greater following exposure to labeled females (Fig 3B).

We then modified the experiment by labeling males with the dye and placing them with an unlabeled female. We found that the dye was transferred from males to the wing margin of the female (Fig 3C). Finally, we placed labeled males in a chamber with an unlabeled male and found transfer to the wing margin of the recipient male (Fig 3D).

The simplest interpretation of these results is that large hydrophobic compounds of limited volatility can be transferred from one fly to the anterior wing margin of another fly, where chemosensory sensilla reside.

### *IR52a* males are defective in sexual behavior

Based on its expression pattern, its coexpression with *fru*, and its predicted 44% amino acid sequence identity to both *IR52c* and *IR52d*, with which it is clustered in the genome (S7A Fig), we wondered if *IR52a* was required for normal sexual behavior. We used CRISPR/Cas9 technology to generate a deletion lacking most of the *IR52a* coding sequence. The deletion removes two of the three predicted transmembrane domains and the predicted ligand binding domains but does not extend beyond the *IR52a* coding region (S7B Fig). We backcrossed deletion lines for five generations against our control line to minimize the possibility of genetic background effects.

In *D*. *melanogaster*, a complex courtship ritual precedes copulation. One element of this ritual is the extension of a male wing to produce a courtship song via wing vibration [49–52]. To quantitate courtship behaviors, we used an updated version of FlyVoyeur, which allows automated tracking of flies and classification of their behavior during courtship [15].

**Fig 4:**
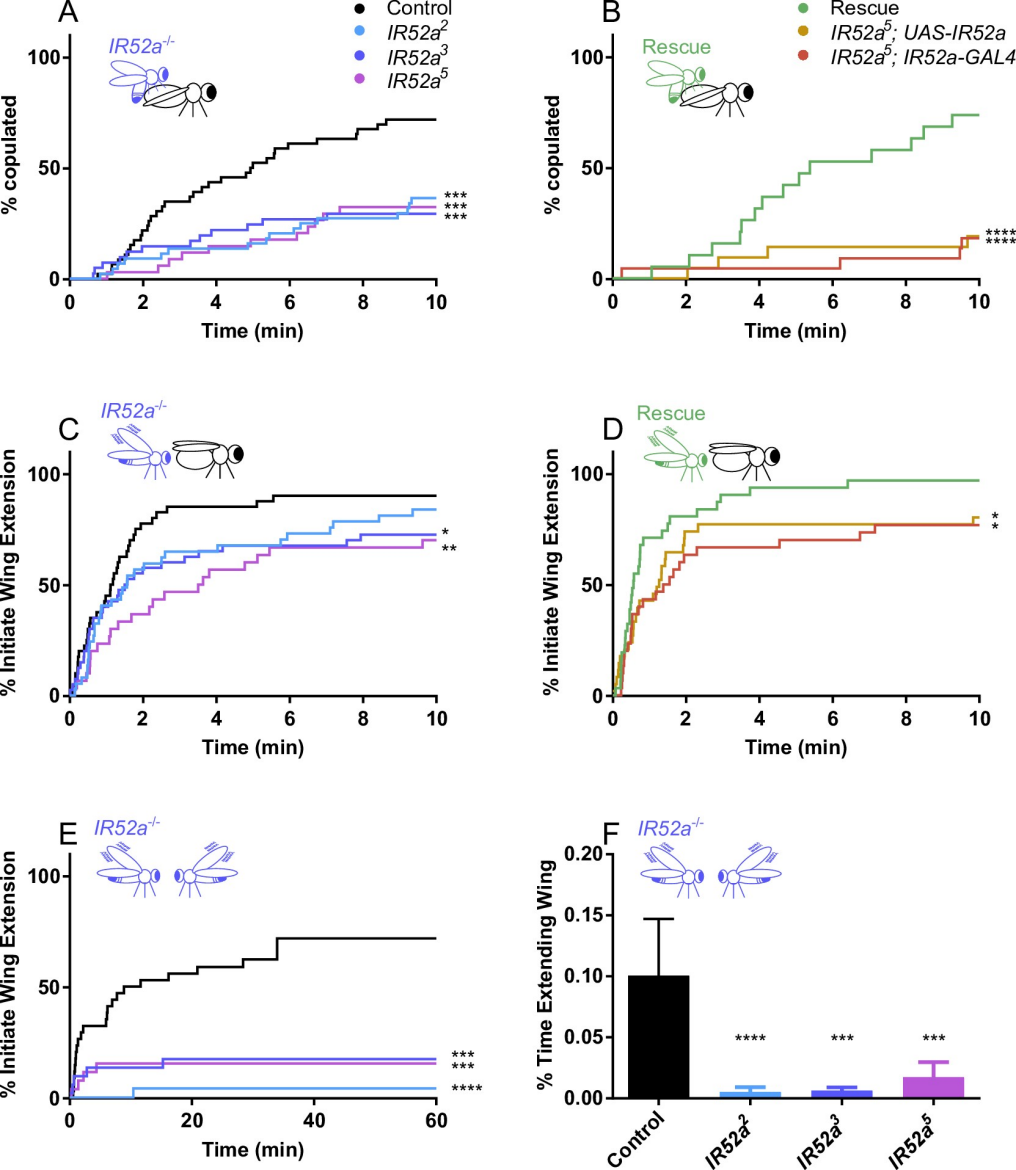
*IR52a* males are defective in sexual behavior. (A) *IR52a* males show delayed and reduced copulation with *Canton-S w*^*1118*^ females. ***P < 0.001 compared to control *w*^*1118*^ males (log-rank test). *n* = 34–46 for each genotype. (B) Rescue of *IR52a* mutant male phenotype. The rescue construct was a *UAS-IR52a* cDNA that had been backcrossed to the *w*^*1118*^ background five times, as had the *IR52a-GAL4* construct. The genotype of the rescued flies is *IR52a*^*5*^; *UAS-IR52a/ IR52a-GAL4*, *n* = 30–32. (C) *IR52a* mutant males showed delayed courtship initiation, *n* = 34–46. For *IR52a*^*2*^, *P* = 0.08. (D) Rescue of *IR52a* mutant male phenotype. Genotypes are the same as in panel B, *n =* 30–32. (E) *IR52a* mutant males showed reduced courtship initiation toward other males, *n* = 26–32. The *IR52a*^*3*^ values are nudged +2% on the y-axis to avoid overlapping with the *IR52a*^*5*^ values. (F) *IR52a* males showed reduced courtship toward other *IR52a* males. Mean +/− SEM, *n* = 26–32. ***P < 0.001; ****P < 0.0001 compared to control, Kruskal–Wallis test, Dunn's multiple comparisons test. In (A–E), *P < 0.05; **P < 0.01; ***P < 0.001; ****P < 0.0001, log-rank test. Underlying data for this figure can be found in S1 Data. IR, ionotropic receptor.

When *IR52a* mutant males were paired with virgin females in a chamber, they showed a delay in achieving successful copulation (Fig 4A). This phenotype was observed with each of three independent alleles. Specifically, by the time 50% of control males had begun to copulate, only approximately 20% of mutant males had initiated copulation. Moreover, at the end of the observation period, only half as many mutant males as wild-type males had copulated.

To determine whether this phenotype in fact arose from the lack of *IR52a*, we performed a rescue experiment (Fig 4B). We found that mutant males expressing an *IR52a* rescue construct with an *IR52a-GAL4* driver initiated copulation much faster than control males.

We also measured the time until the first male wing extension event and plotted the percentage of males that had shown such an initial event as a function of time. Two of the three alleles showed a significant delay (Fig 4C, *P* < 0.05); the third allele showed a delay that was not significant (Fig 4C, *P* = 0.08). We attempted to rescue the phenotype of one allele, *IR52a*^*5*^, and found that expression of the *IR52a* transgene did in fact accelerate wing extension relative to either control (Fig 4D, *P* < 0.05).

Finally, we asked whether *IR52a* affects male–male courtship behavior. Males show much less wing extension toward other males than toward virgin females, but extensions can be observed and quantitated in two ways. First, we measured the time to the first wing extension and found that wing extension was severely reduced in *IR52a* males relative to control males; in fact, only a small fraction of mutant males had shown a wing extension event by the end of a 60 minute observation period (Fig 4E). Second, we measured the fraction of the entire observation period during which males were engaged in wing extension. Pairs of *IR52a* mutant males extended their wings for a much shorter aggregate time than pairs of control males (Fig 4F).

To test whether the defects in sexual behavior might be due to general defects in mobility or health, we carried out tests of motor function and found no abnormalities in *IR52a* males or females (S8 Fig).

### Optogenetic silencing of *IR52a* neurons inhibits sexual behavior in males

To explore the role of *IR52a* in more detail, we used optogenetics to silence the neurons in which it is expressed. We used *IR52a-GAL4* to drive expression of the anion channelrhodopsin GtACR1 [53] and then tested behavior of male flies that were or were not fed the chromophore all-trans-retinal (ATR).

**Fig 5:**
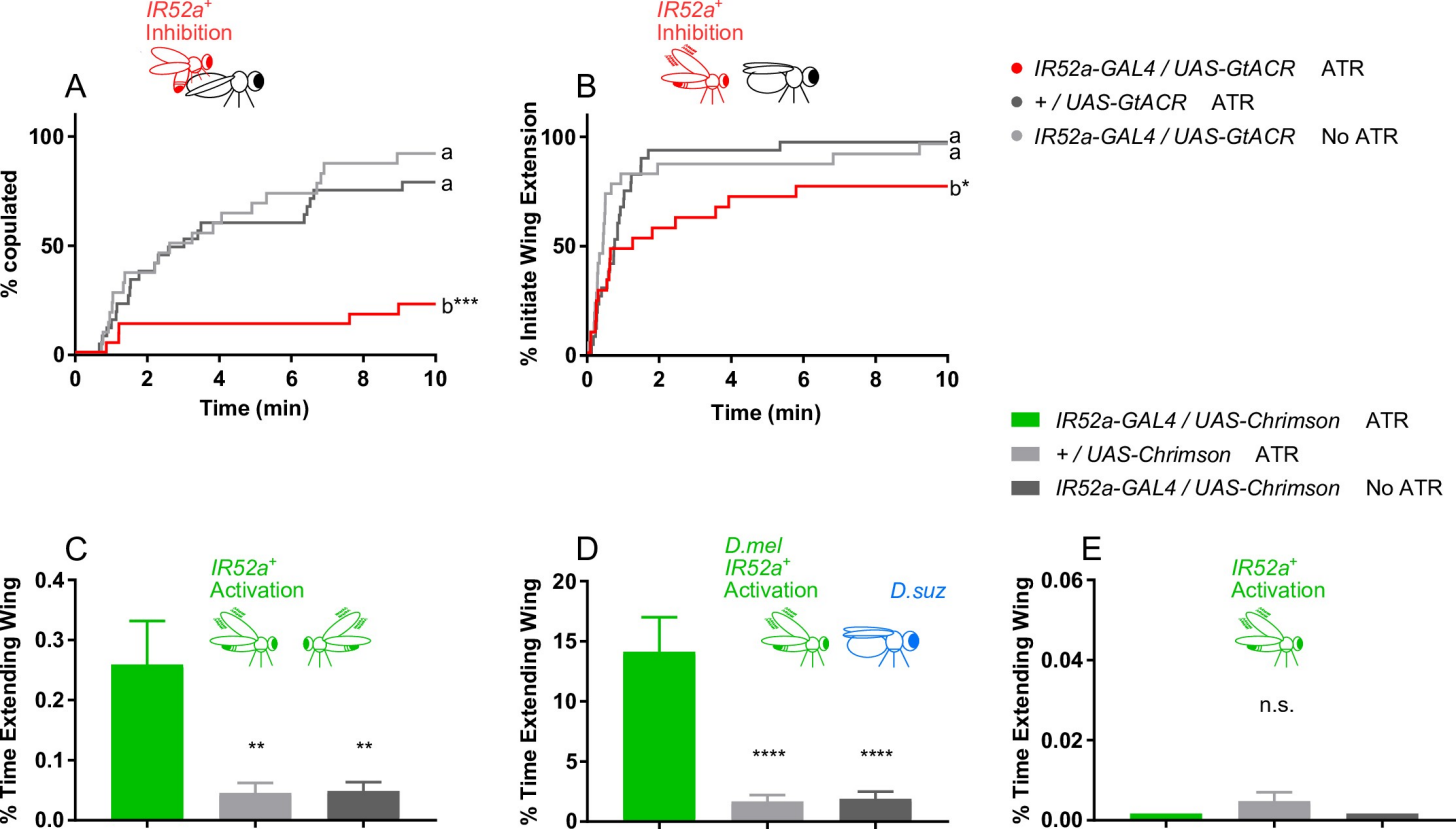
Silencing or activating IR52a^+^ neurons in males affects sexual behavior. (A) Inactivation of IR52a^+^ neurons in males reduces copulation. ***P < 0.001 compared to control, log-rank test, *n* = 23–27 for each genotype. (B) Inactivation of IR52a^+^ neurons reduces courtship initiation. *P < 0.05 compared to control, log-rank test, *n* = 23–27. (C) Activation of IR52a^+^ neurons promotes male–male courtship behaviors. **P < 0.01, ANOVA, Holm-Sidak’s multiple comparisons test, *n* = 22–28. (D) Activation of IR52a^+^ neurons promotes *D*. *melanogaster* male courtship behaviors toward *D*. *suzukii* females. ****P < 0.0001, ANOVA, Holm-Sidak’s multiple comparisons test, *n* = 20–21. (E) Activating IR52a^+^ neurons in solitary males did not induce wing extension. All data points were 0 in the first and third columns. ANOVA, one-sample *t* test against 0 for the second column; unavailable for the first and third column, *n* = 22–24 for all genotypes. Underlying data for this figure can be found in S1 Data. IR, ionotropic receptor; ns, not significant.

When exposed to continuous red light during the testing period, *IR52a-GAL4; UAS-GtACR* males that had been fed ATR showed a major reduction in copulation success compared to control males (Fig 5A). There was also a decrease in the initiation of wing extension (Fig 5B). The results of this optogenetic experiment indicate that the role of *IR52a* is physiological, as opposed to developmental.

### Activation of *IR52a* neurons drives sexual behavior beyond the species barrier

The optogenetic inhibition studies indicate that the IR52a^+^ neurons are required for male courtship. To determine whether IR52a^+^ neurons drive a circuit that promotes courtship behavior, we carried out optogenetic activation studies with the excitatory channelrhodopsin Chrimson under red light.

As a sensitive assay, we tested the effects of activating IR52a^+^ neurons in circumstances in which control activity levels are low. We first paired males with other males. Whereas levels of wing extension between control males are low, levels of wing extension between *IR52a-GAL4; UAS-Chrimson* males fed ATR are dramatically higher (Fig 5C).

We then paired males with females of a distantly related species, *D*. *suzukii*. Control *D*. *melanogaster* males showed little wing extension toward *D*. *suzukii* females. Remarkably, when IR52a^+^ neurons are activated, the fraction of time spent in wing extension was an order of magnitude higher, approaching 15% (Fig 5D).

Finally, we asked whether activation of IR52a^+^ neurons would generate wing extension behavior in the absence of a target fly and found that it did not (Fig 5E). These results are consistent with the expectation that a male requires additional cues in order for input from IR52a^+^ neurons to activate sexual behavior.

### *IR52a* is required for sexual behavior in females

*IR52a* is expressed in both males and females (S3 Fig and S4 Fig) [15]. We wondered whether *IR52a* also played a role in female sexual behavior. To test this possibility, we paired *IR52a* females with *IR52a*^*+*^ males and measured copulation latency.

**Fig 6:**
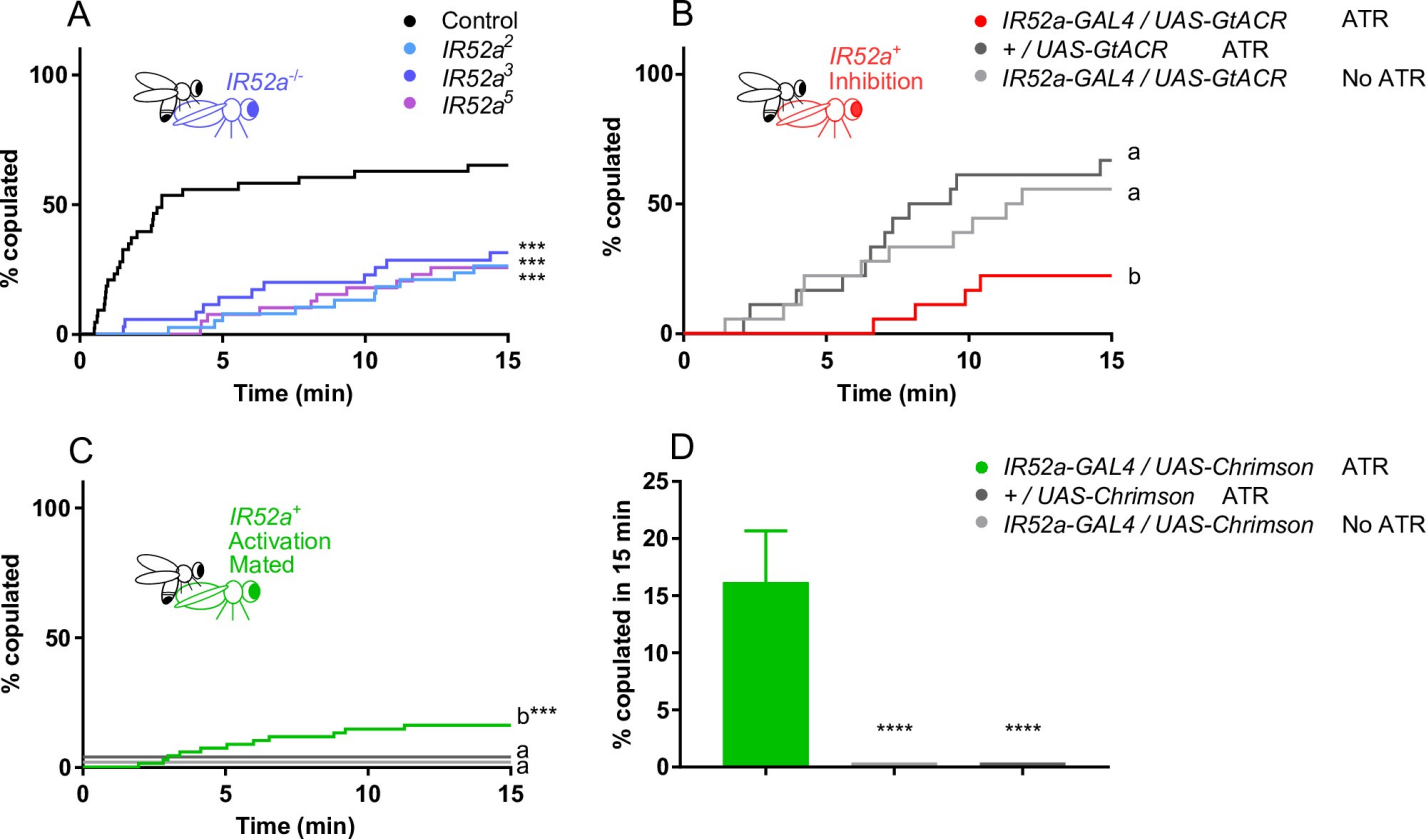
*IR52a* females showed reduced copulation. (A) *IR52a* females show reduced copulation. ***P < 0.001 compared to control *w*^*1118*^ females, log-rank test, *n* = 35–43. (B) Inactivation of IR52a^+^ neurons reduces female copulation. p<0.01 for b versus *+/UAS-GtACR* ATR; *P* < 0.05 for b versus *IR52a-GAL4/UAS-GtACR* no ATR, log-rank test, *n* = 18. (C) Activation of IR52a^+^ neurons promotes mated *D*. *melanogaster* female copulation. *P < 0.05, log-rank test, *n* = 32. The *+/UAS-Chrimson* ATR values are nudged +4% and the *IR52a-GAL4/UAS-Chrimson* No ATR values are nudged +2% on the y-axis to avoid overlapping with the x-axis. (D) Activation of IR52a^+^ neurons promotes mated *D*. *melanogaster* female copulation. The graph shows copulation at 15 min; data taken from panel (C). ****P < 0.0001, Kruskal–Wallis test, Dunn's multiple comparisons test. Underlying data for this figure can be found in S1 Data. ATR, all-trans-retinal; IR, ionotropic receptor.

*IR52a* females showed a prolongation of the time to copulation, and by the end of the test period, the number of copulating *IR52a* females was much lower than the control value (Fig 6A). The same female phenotype was observed for all three alleles of *IR52a*.

We asked whether *IR52a* females elicited less wing extension from males than did control females. Male wing extension to these mutant females was not reduced (S9 Fig). One simple interpretation of these results is that in wild-type females, IR52a detects a pheromonal signal from males that makes the females more receptive to the males.

To our knowledge, there are few other receptor genes besides *IR52a* that are required for both male and female sexual behavior in *Drosophila* [27].

### Optogenetic manipulation of IR52a^+^ neurons in females affects sexual behavior

We tested the effects of inactivating IR52a^+^ neurons in females. When IR52a^+^ neurons were inactivated by *GtACR*, copulation was reduced (Fig 6B). The magnitude of the reduction was comparable to that observed following deletion of the *IR52a* gene (Fig 6A).

Finally, we tested the effects of activating IR52a^+^ neurons in mated females. Mated females normally show much less copulation than virgin females due in part to factors that are transmitted from the male to the female during copulation and that reduce her receptivity [54, 55]. This mating suppression provides a sensitive assay for factors that increase female receptivity. An increase in copulation was in fact observed in mated *IR52a-GAL4; UAS-Chrimson* females that had been fed ATR and illuminated with red light compared to control females: 19% of the activated females copulated versus 0% in both controls (Fig 6C).

Taken together, these activation and inactivation experiments suggest that in females, IR52a^+^ neurons receive a male signal that acts to promote female receptivity. A simple interpretation of our experiment with mated females is that optogenetic activation of IR52a^+^ in these females was sufficient to partially override the mating suppression.

### Trans-synaptic mapping of second-order neurons

We used a newly developed method for circuit mapping, *trans-*Tango, to identify postsynaptic partners of IR52a^+^ neurons [56]. Briefly, the system uses a synthetic signaling pathway that is introduced into the fly. We used *IR52a-GAL4* to drive expression of myrGFP and one component of the pathway, a membrane-bound ligand, in IR52a^+^ neurons. This membrane-bound ligand is designed to bind to a receptor in postsynaptic partners and cause them to express mtdTomato. All neurons of the fly contain the receptor, but in principle, only neurons postsynaptic to the IR52a^+^ neurons are induced by the membrane-bound ligand to express mtdTomato. In this manner, IR52a^+^ neurons are labeled with GFP, and candidate second-order neurons are labeled with mtdTomato.

**Fig 7:**
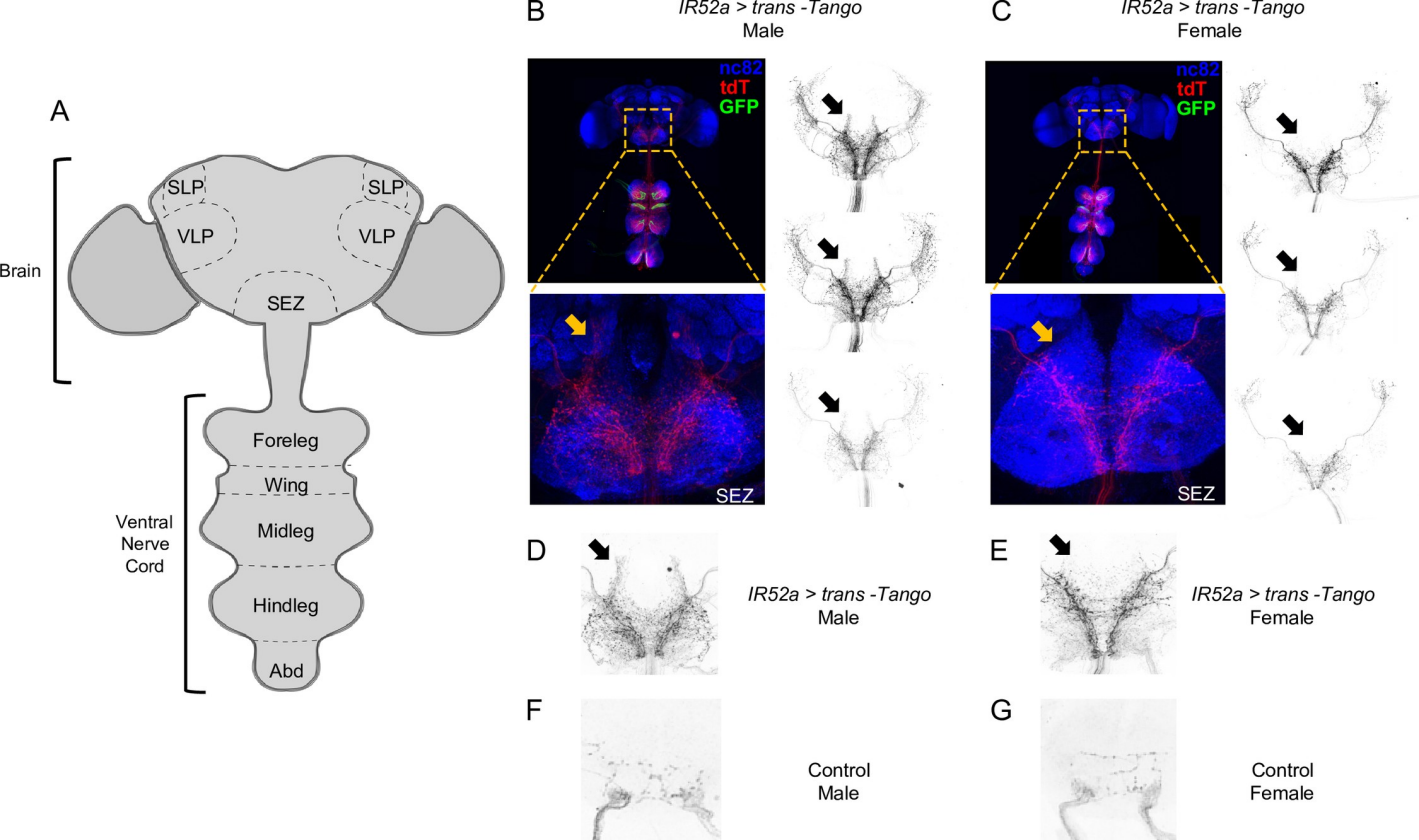
*IR52a*^*+*^ neurons synapse with sexually dimorphic second-order neurons. (A) The *Drosophila* CNS. (B) Expression pattern of *IR52a* > *trans*-Tango in male flies. Green, *IR52a*^*+*^ sensory neurons; red, candidate second-order neurons labeled with mtdTomato (tdT); blue, neuropil (nc82 labeled with an anti-Bruchpilot antibody). Orange and black arrows indicate sexually dimorphic neurites in colored and black-and-white images from different flies. (C) Expression of *IR52a* > *trans-*Tango in female flies. Note the lack of neurites in the areas indicated by the arrows. (D) Higher magnification of the SEZ, showing labeling of the candidate second-order neurons in male flies. The arrow indicates a sexually dimorphic region. (E) Labeling of the candidate second-order neurons in the SEZ in female flies. (F) Control male flies with *trans-*Tango components but without the *IR52a-GAL4* driver. (G) Control female flies. CNS, central nervous system; GAL4; IR, ionotropic receptor; SEZ, subesophageal zone; SLP, superior lateral protocerebrum; VLP, ventrolateral protocerebrum.

As expected, when we examined the central nervous system (CNS) of flies expressing *IR52a-GAL4* and the components of the *trans*-Tango system, we observed GFP-labeled axons in the ganglia of the ventral nerve cord (VNC), which receives input from the wing and legs (green in Fig 7A–7C). We observed mtdTomato signal in both the VNC and in the brain. Labeling in the brain is sparse but robust, with axon terminals arborizing in the subesophageal zone (SEZ) and also in the ventrolateral protocerebrum (VLP) and superior lateral protocerebrum (SLP). Interestingly, the mtdTomato labeling in the SEZ is sexually dimorphic, as illustrated by the presence of neurites that are labeled in males (note the five examples indicated by arrows in Fig 7B and 7D) but that are absent or show very little labeling in females (Fig 7C and 7E).

**Fig 8:**
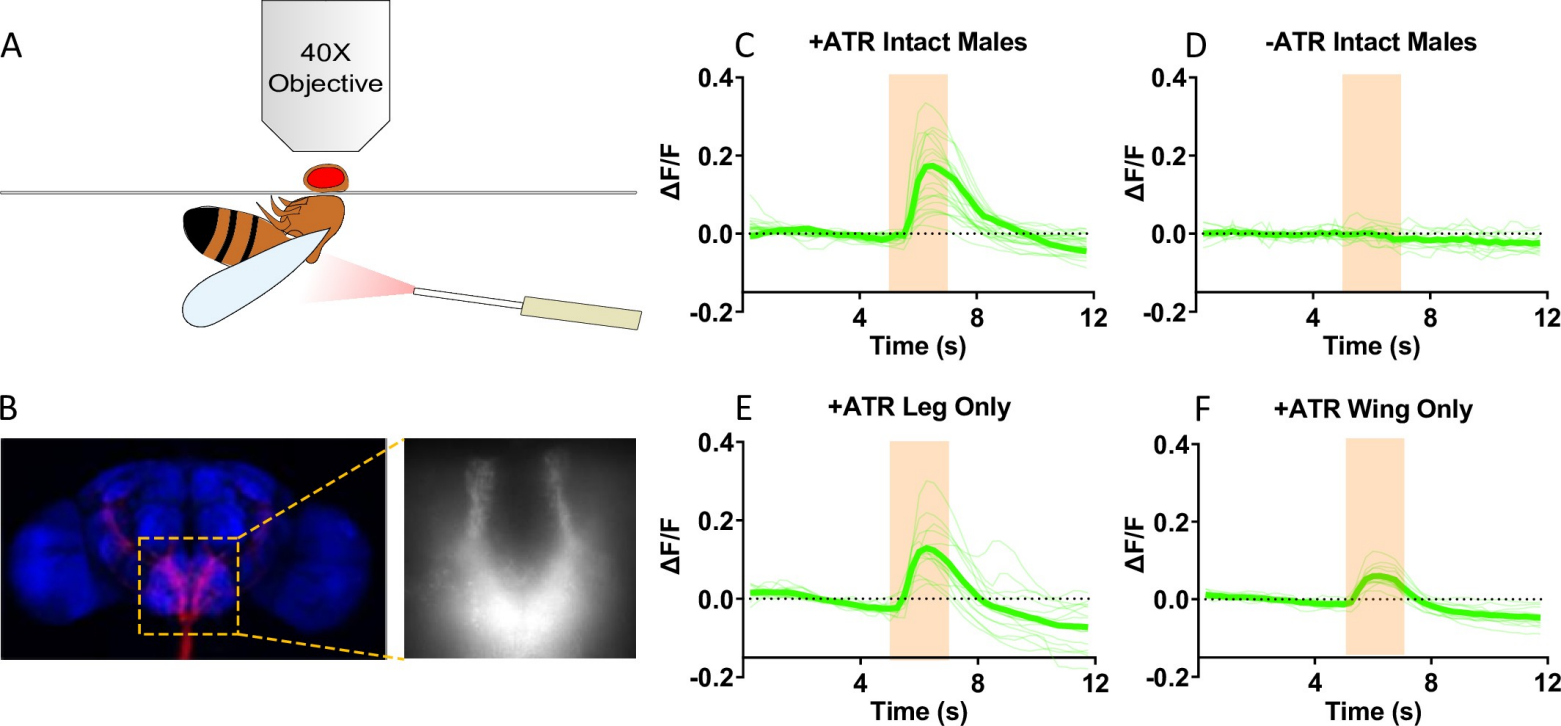
Leg and wing *IR52a*^*+*^ neurons form functional connections with neurons in the SEZ. (A) The calcium imaging setup. The optic fiber is placed 0.5 cm away from the side of the thorax of a male fly. The proboscis and head cuticle were removed to expose the SEZ. The stimulation wavelength is 617 nm. (B) Example images of the region in the SEZ. (C) ReaChR-mediated optogenetic activation of *IR52a*^*+*^ neurons triggers a Ca^2+^ increase in the SEZ, visualized by GCaMP3 imaging in flies fed on ATR food, *n* = 24. Orange shading indicates the stimulation period. Individual trials were plotted with thin green lines, and the average signal was plotted with a thick green line. (D) No calcium signal was observed in the SEZ in flies fed on regular cornmeal without ATR, *n* = 14. (E) ReaChR-mediated optogenetic activation of leg *IR52a*^*+*^ neurons triggers a Ca^2+^ increase in the SEZ. Wings were removed bilaterally 3 days prior to illumination and imaging, *n* = 14. (F) Activation of wing IR52a^+^ neurons triggers a Ca^2+^ signal in the SEZ. The tarsal segments and tibia of all six legs were removed 3 days prior to illumination and imaging, *n* = 13. Underlying data for this figure can be found in S1 Data. ATR, all-trans-retinal; IR, ionotropic receptor; ReaChR; SEZ, subesophageal zone.

To confirm the functional connectivity between IR52a^+^ neurons and the second-order neurons revealed by *trans*-Tango, we expressed the red-shifted channelrhodopsin ReaChR in IR52a^+^ neurons and GCaMP3 instead of mtdTomato in the candidate second-order neurons, as driven by the *trans*-Tango system. When red light was delivered toward intact males raised on food supplemented with the chromophore ATR, a robust Ca^2+^ signal was observed in the SEZ (Fig 8A–8C). No signal was observed when flies were raised in the absence of ATR (Fig 8D).

To test whether these synapses are formed with axons from the legs, wing, or both, we carried out ablation experiments. We ablated the tarsal segments and tibia of all six legs or, in a parallel experiment, both wings. We found that 3 days is sufficient for the axons of IR52a^+^ neurons to degenerate (S10 Fig). After degeneration, we delivered red light toward the flies. We detected Ca^2+^ signal in the SEZ of flies that had legs but not wings (Fig 8E) and in flies that had wings but not legs (Fig 8F). These results support the notion that both leg and wing axons transmit chemosensory information from IR52a receptors to the SEZ.

Finally, we attempted to confirm these results via ablation experiments in flies engineered to allow *trans*-Tango labeling. We ablated the tarsi and tibia of the legs on one side of a fly, allowed the axons to degenerate, and examined the flies 2 weeks later. As expected, GFP labeling disappeared from the leg neuropils on the ipsilateral side of the VNC (S11 Fig; see Fig 7A for map). In the brain, the level of mtdTomato in the ipsilateral side relative to the contralateral side declined, such that ipsilateral labeling was only approximately 85% that of contralateral labeling (S11A–S11C Fig).When both the wing and legs were ablated on the ipsilateral side, there was a larger decline in GFP labeling on the ipsilateral side (*P* = 0.001, *t* test), indicating that much of the labeling on the ipsilateral side was due to projections from wing axons. We also observed a decline when labeling was quantitated only in the SEZ (S11D Fig), providing further evidence that IR52a^+^ wing axons have postsynaptic partners in the SEZ. Moreover, the SEZ labeling was qualitatively similar on the two sides following each kind of ablation, supporting the notion that there is a good deal of similarity between the SEZ projections from the wing and legs.

## Discussion

We have carried out a molecular analysis of a greatly understudied taste organ of the fly. Through a differential RNA-seq screen, we identified genes expressed in chemosensory sensilla of this organ. One of these genes, *IR52a*, is required for both male and female mating behavior. Optogenetic analysis of IR52a^+^ neurons has provided new insight into the molecular logic of sexual behavior.

### Chemosensory sensilla of the wing: A molecular profile

After confirming that the curved sensilla on the wing margin contain pores, we examined their molecular organization by transcriptional profiling. We identified 650 genes expressed at higher levels in wings that contain chemosensory sensilla than in wings that do not. This differential screening identified a roster of chemosensory genes that are likely to form the molecular underpinnings of chemoreception in the wing.

As a byproduct, the analysis also identified a number of other interesting genes—including *IRs*, *Grs*, *ppks*, and *Obps*—that are also expressed in the wing but likely in other kinds of sensilla or cells. Some of these genes may provide a focus for the study of other wing functions, such as mechanosensation.

We note that many of the wing genes identified in our analysis, including both genes that are and are not enriched in chemosensory sensilla, were also recently identified in a different kind of RNA-seq comparison: between the entire wing of *Drosophila* and the wing and gut of a distantly related aphid, *Acrytosiphon pisum* [57]. In addition to a number of *Obp*, *Che*, and *ppk* genes, *IR52a* was among 12,188 genes found by that study to be expressed in the *Drosophila* wing, in agreement with our findings.

It is striking that no *Gr* genes were identified by our differential screen as candidate wing chemosensory receptors. A priori, one might have expected taste sensilla of the wing to express a variety of receptors of this ancient family. However, the dearth of *Gr* expression is consistent with a previous finding: few, if any, of 67 *Gr-GAL4* drivers, representing the entire repertoire of Gr taste receptors, labeled axons projecting to the wing neuropil of the VNC [58].

By contrast, we found a variety of *IR* and *ppk* transcripts to be enriched in the chemosensory sensilla. IR25a and IR76b, the two most abundantly expressed IRs identified, have been shown to act as coreceptors for other IRs in other contexts [13]. The Ppk receptors include Ppk23, Ppk25, and Ppk29, which have previously been implicated in pheromone reception in other taste organs but which have also been found expressed in the wing [22–26]. These results validate our screen and support the possibility of a role for the other Ppks we identified, such as Ppk10.

The number of identified Obps and Che proteins was surprising: 14 and 11, respectively. Either an individual chemosensory sensillum coexpresses many of these proteins or else the wing contains multiple types of chemosensory sensilla, each with a distinct molecular profile. Four of these Obps are enriched in gustatory sensilla of the labellum, and one of them is required for normal response to taste mixtures [59]. There was little overlap between these 14 wing Obps and the 10 most abundant Obps of the antenna [60].

We note finally that we have identified candidate chemosensory genes by virtue of their expression in control wings but not *poxn* wings that lack chemosensory sensilla. While the simplest interpretation of this differential expression is that the genes are expressed in chemosensory sensilla, the expression of any individual gene in these sensilla needs confirmation. It is formally possible that *poxn* has unexpected, indirect effects that repress transcription in other cell types of the wing.

### Wings as recipients of cues

Wings have long been known to produce sexual cues: the male wing vibrates to generate a courtship song [52, 61]. Here, we have shown through dye transfer experiments that wings are also likely to receive sexual cues.

The wing margin offers anatomical advantages as a sensor of chemical information. It serves as the lateral boundary of the fly and provides an extensive zone for contact with other flies and the environment. Moreover, the wing margin may receive chemical information indirectly via grooming: the legs of a fly may receive chemical cues from other flies or the environment and then transfer these cues to its wings.

The notion that wing chemosensory sensilla receive sexual information is consistent with their expression of *fru*. The *fru* gene specifies central neural circuits that influence male sexual behaviors [46–48, 62]. *fru* is also expressed in peripheral neurons of the leg that are required for sexual behavior [51] and is coexpressed in these neurons with the pheromone receptor Ppk23 [23]. The coexpression of *fru* with *IR52a* in wing neurons may reflect a role for these neurons in sexual behavior as well.

### A new role for an *IR* in regulating male and female sexual behavior

Expression of *IR52a* in the wing is supported by four lines of evidence: RNA-seq, RT-PCR, qPCR, and *GAL4* driver analysis. Expression is found in both male and female wings, in both *D*. *melanogaster* and in *D*. *suzukii*. *IR52a* is also expressed in both male and female leg sensilla [15]. The expression in both sexes is consistent with our finding that *IR52a* is required in both males and females for normal sexual behavior. To our knowledge, few receptor genes have been shown to be required for the sexual behavior of both males and females.

The requirement for *IR52a* for male sexual behavior toward females was confirmed by the finding from optogenetic analysis that the activity of IR52a^+^ neurons is required as well. In fact, the phenotype produced by deleting the gene was similar to that produced by silencing the neurons (Figs 4A, 4C, 5A and 5B). This similarity in phenotype suggests that the contribution of IR52a^+^ neurons to male behavior toward females depends primarily on the activity of IR52a.

In males, the loss of *IR52a* reduces the response to both females and males. One function of IR52a might be to act in the detection of a pheromone produced by both females and males. *Drosophila* produces a wide variety of cuticular hydrocarbons that are candidate pheromones, some of which are male-specific, some female-specific, and some common to both [32–34, 63]. There is precedent for pheromones that are detected by both males and females of a species [64]. Such pheromones could act in species recognition.

IR52a may also act in the detection of sex-specific pheromones. IRs have been shown to form heteromultimers [12, 65]. We have found that chemosensory sensilla express other IRs, including other IRs in the IR52 cluster, in both wings and legs (S1 Fig and [15]). It seems plausible that IR52a may form different heteromers that respond to different pheromones in different neurons. This kind of combinatorial logic could allow a limited number of receptors to detect a larger number of pheromones. Perhaps different heteromers form in males and females, conferring responses to a female or male pheromone, respectively. It is possible that IR52a functions as a coreceptor.

Optogenetic experiments show that once activated, IR52a^+^ neurons drive *D*. *melanogaster* male courtship behavior, even to unproductive targets such as males and *D*. *suzukii* females, which diverged from *D*. *melanogaster* on the order of 25 million years ago [66–69]. Evidently, strong IR52a^+^ activation overrides the need for cues specific to *D*. *melanogaster* females. However, other cues are still required: a lone male does not show wing extension in the absence of a fly target. The input from IR52a^+^ neurons may need to be integrated with input driven by other sensory cues, perhaps visual cues, in order for courtship behavior to proceed.

Optogenetic inactivation and activation of IR52a^+^ neurons in females also decreased and increased levels of copulation, respectively. The level of copulation is expected to depend on the degree of female receptivity, which in turn is influenced by a variety of factors [70, 71]. The simplest interpretation of our results is that the level of activity of female IR52a^+^ neurons is one such factor. IR52a^+^ activation may increase the receptivity of females and thereby drive higher copulation levels. In any case, the results with females provide further evidence that IR52a^+^ neurons are elements of the circuitry that promotes sexual behaviors.

As an initial step in mapping the circuitry, we used the *trans*-Tango method to show that IR52a^+^ neurons connect with neurons that project to the SEZ as well as the VLP and the SLP (Fig 7). Moreover, Ca^2+^ imaging of the SEZ supports the notion that many if not all of these connections are functional synapses (Fig 8). Interestingly, some of these projections are sexually dimorphic: easily detectable in males but not females. The sexual dimorphism is consistent with a role for these neurons in conveying pheromonal information, which may be routed differently in the CNS of males and females [72]. Ca^2+^-imaging experiments (Fig 8) and ablation experiments (S11 Fig) each indicated that information from both wings and legs is relayed to the SEZ.

We note that several gustatory projection neurons have been described previously. TPN2 neurons have axonal termini in the SEZ and in the higher brain and respond to sucrose [73]. vAB3 neurons have projections that terminate in the SEZ and in the lateral protocerebral complex; they are sexually dimorphic and respond to pheromones [74, 75]. Pheromone Projection Neuron 1 (PPN1) neurons send projections to the VLP [76]. It will be interesting in the future to examine the anatomical relationship between these neurons and those described here, for example, through split-GAL4 analysis.

The simplest interpretation of all of our results, taken together, is that IR52a functions as a pheromone receptor in the leg and the wing. We cannot exclude, however, the formal possibility of a more complicated model in which IR52a acts as a pheromone receptor in the leg but as a receptor for another ligand in the wing. However, even in this case, it seems likely that the information from the two signals would be integrated in the CNS based on the qualitative similarity of SEZ projections from wing and leg, as evidenced by *trans*-Tango results.

In summary, IR52a is a new element in the logic that controls sexual circuits in the fly. It is unusual in that it acts in both males and females and that it can promote both male–male and male–female interactions. Moreover, IR52a^+^ neurons can override the circuits that normally suppress sexual behavior toward unproductive targets. The ability of IR52a^+^ neurons to drive sexual behavior beyond sex and species barriers opens new avenues of investigation into the interactions among sexual circuits that are activated by different sensory cues.

## Materials and methods

### *Drosophila* stocks

Flies were reared on corn syrup and soy flour culture medium (Archon Scientific) at 25°C and 60% humidity in a 12:12-hour light–dark cycle, except *trans*-Tango flies were raised at 18°C. *IR52a-GAL4* and *IR52a-LexA UAS-mCD8GFP LexAop-mtdTomato* were described in [15]. *UAS-GtACR1* was a gift from Adam Claridge-Chang [53]. *Poxn*^*70*^ was described in [35]. *UAS-CsChrimson* was a gift from Vivek Jayaraman and was described in [77]. *trans*-Tango line was from Bloomington *Drosophila* Stock Center (#77124) and was described in [56]. *IR52a* mutations are described below. All lines were backcrossed to Canton-S *w*^*1118*^ (stock 0408–1) for at least five generations.

### *IR52a* deletions

*IR52a* deletions were generated using CRISPR/Cas9 homologous recombination. Guide RNAs (gRNAs) were designed using the flyCRISPR Optimal Target Finder [78]. gRNA plasmid was constructed using pCFD4 (Addgene 49411), following the protocol described in [79]. Gibson Assembly was performed using Gibson Assembly Master Mix (New England BioLabs, E2611S). gRNA plasmid and a donor plasmid (pDsRed-attP, Addgene 51019) with homologous arms and a DsRed marker was injected into embryos by Bestgene, Inc. (Chino Hills, CA). DsRed positive alleles were then backcrossed to our control *w*^*1118*^ Canton-S line for five generations. The primers used in the construction are shown in S7C Fig.

### Optogenetic activation and inactivation

Flies were collected on the first day of eclosion and were given fly food containing 0.5 mM ATR. Flies were kept in the dark for 3–4 days before testing in behavioral assays. During the assays, the arena was illuminated with a custom-built LED matrix with a wavelength of 625 nm (SMD 5050, LEDlightninghut.com) at an intensity of 1.75 +/− 0.11 W/m^2^. We did not observe neuronal death after long light exposure: the CsChrimson we used is tagged with EYFP, and the morphology of EYFP^+^ neurons remained grossly normal after 15 minutes of exposure.

### Scanning electron microscopy

Fly wings were dissected and mounted on 9.5 mm aluminum stubs (Electron Microscopy Services #75180) using carbon paint (Electron Microscopy Services #12691–30). Samples were coated with 8 nm iridium with a Cressington 208 iridium sputtering tool. Microscopy was carried out with a Hitachi SU-70 electron microscope equipped with solid-state backscatter detector for enhanced imaging of grain boundaries.

### Confocal imaging

Images of GFP and Tomato labeling were acquired on Zeiss LSM880 and LSM510 confocal microscopes and processed using Fiji (ImageJ 1.51n) [80].

### RNA isolation and RNA sequencing

The wings from *w*^*1118*^ Canton-S and *poxn* flies aged 3 days were hand-dissected and frozen immediately in liquid nitrogen. Three independent replicates were collected per genotype, each replicate containing 8000 wings, from 4000 flies. RNA extraction was performed using an RNeasy Mini Kit (QIAGEN 74104) with on-column DNase treatment (QIAGEN 79254). Total RNA was sent to myGenomics, LLC (Alpharetta, GA). Poly(A)^+^ RNA was selected and fragmented, and samples were prepared for double-end mRNA sequencing (PE100) with standard protocols. The total analysis included 60 million reads per sample.

### RNA sequencing data analysis

Trim Galore! (0.4.0) was used to perform quality control and adapter trimming on the raw reads. STAR (2.4.2a) was then used to align reads to the *D*. *melanogaster* genome (FlyBase release r6.09) [81]. The differential analysis was performed using DESeq2 [36]. An volcano plot was made by DESeq2 and custom R script. Gene ontology enrichment analysis was done using PANTHER [82].

Raw sequencing reads and preprocessed results are available from the Gene Expression Omnibus (GSE115815).

### RT-PCR

Wings (approximately 100 pairs for each sex) were dissected from 3-day old virgin flies using forceps and placed immediately into Eppendorf tubes kept cold in liquid nitrogen. For total RNA extraction, tissues were kept in RLT buffer (QIAGEN) with 1% β-mercaptoethanol, crushed with RNase-free plastic pestles first, and then sonicated 10 times, for 10 seconds each time at 4°C. After sonication, total RNA was extracted with a QIAGEN RNeasy kit. mRNAs were treated with RNAse-free DNase I (QIAGEN #79254) before being used for first strand cDNA synthesis with Superscript RT III (Invitrogen), followed by PCR amplification with 35 amplification cycles. To control for contamination by genomic DNA, each batch of mRNA underwent a parallel mock reverse transcription step (no RT control) in which the reverse transcriptase was omitted, before being subjected to PCR. To provide a semi-quantitative comparison of RNA quality and quantity between samples, an RT-PCR product of eIF1α was electrophoresed on agarose gels next to the *IR52a* RT-PCR products. PCR primers are listed below:

*eIF1α* forward: AGCCCACCAATATGATGTCG

*eIF1α* reverse: CTTCAAGGAGGACCAACAGG

*IR52a* forward: TCCTGCTGAACGACAAGAGC

*IR52a* reverse: GGCTGAGTGAAATACGACTGC

### Quantitative RT-PCR

Three-day-old virgin flies were used in RNA extraction as described above. Quantitative PCR was performed in a CFX96 TouchTM real-time PCR detection system (Bio-Rad). cDNA from the RT reaction and the No-RT control solution were each mixed with iTaq Universal SYBR Green Supermix (Bio-Rad #1725121). The denaturation temperature was set to 95°C, annealing and extension temperatures were set to 60°C. Two different pairs of primers were used for *IR52a* qPCR. A standard curve was made to ensure optimum primer efficiency and a melting curve was generated to ensure specificity. PCR primers are listed below:

*IR52a* fwd A: TCCTGCTGAACGACAAGAGC

*IR52a* rev A: GGCTGAGTGAAATACGACTGC

*IR52a* fwd B: AACGGTGGCAGATAGTTTGG

*IR52a* rev B: CATTCAGTTTCTGGGCATAGG

*eIF1α* fwd: AGCCCACCAATATGATGTCG

*eIF1α* rev: CTTCAAGGAGGACCAACAGG

### Mating assays and automated scoring of behavior

Male flies were collected within 6 hours after eclosion and singly housed until 3–4 days old. Females were collected within 6 hours and aged for 3–4 days on regular fly food in groups of 10 female flies per culture vial. For experiments with mated females, one female was paired with one male immediately after collection. The mating status of the female was confirmed before the behavioral assay by the presence of larvae in the vial.

Mating chambers were built by stacking two multi-well plates (Greiner Bio-One 662000–06) such that each well of the plate was in register with the corresponding well of the other plate. Each chamber was 15.6 mm in diameter and 35.4 mm in height (the sum of the heights of two wells). The wall of each well was coated with Fluon PTFE (BioQuip Insect-A-Slip). A male and female were pipetted into the upper and lower wells, respectively, using a film (3M) to cover each well after the fly had been introduced. After all the individual flies had been introduced into the wells, the film was removed, such that the fly in the upper half of the chamber fell into the lower half of the chamber. The behavior of the two flies was recorded. For optogenetics experiments, the behaviors were recorded at 15 frames/second with cameras (ELP-USBFHD01M-FV), with the infrared filter removed. For recordings under a bright light LED (Gagne 1824-LP-BLACK), a Supereyes B003+ camera was used at 25 frames/second.

The courtship behavior videos were scored using an automated tool custom-written in MATLAB (2015a, MathWorks). We measured unilateral wing extension, which is a signature of courtship; we did not measure bilateral wing extension, which is a signature of aggression and is distinguishable by our algorithm. The source code is available at https://github.com/he-zhe/TwoFlyTracker.

### Dye transfer experiment

Donor flies were labeled in 10-mm x 4-mm diameter chambers that contained a 3-mm diameter filter paper disc attached to the bottom with double-sided tape. We added 15 μL Nile red (Sigma, 2 mg/ml in paraffin oil) to the disc evenly. Then, five flies were labeled by placing them in the chamber for 30 minutes.

Five donor flies and one recipient fly were then transferred to a fresh 10-mm x 8-mm diameter chamber without filter paper or Nile red. After 30 minutes, we froze the chamber to −20°C for 10 minutes to immobilize the flies. Next, the wings of the recipient flies were dissected and observed under an Olympus BX51WP microscope and a CCD camera (QImaging Retiga R3). Excitation light was generated by an Olympus U-LH100HG mercury lamp, and an EGFP bandpass filter set was used (Chroma, 41017). Images were acquired with OCULAR (2.0, QImaging), and then analyzed and quantified using Fiji (ImageJ 1.51n) [80]. The quantification of Nile red in the wing was performed using the measuring tool in Fiji (ImageJ 1.15n). The portion of the wing margin that was anterior to the intersection of the margin and vein LV3 was selected using the segmented line tool, with a line width of 2. The rest of the wing was selected using polygon selections. In both cases, mean fluorescence was recorded, which is independent of the total area.

### Immunohistochemistry and confocal imaging

Immunohistochemistry and fly CNS dissections were performed as described previously [83] with minor modifications. Briefly, flies incubated at 18°C that were 14–21 days old were cold anaesthetized on ice, and dipped into 100% ethanol to make the cuticles less hydrophobic. The flies were then dissected in cold PBS. After 55 minutes fixation of samples in 2% PFA/PBS, samples were washed 4 times for 15 minutes in 0.3% PBST at RT, blocked in 5% Western Blocking Solution (Roche, #11921673001) in 0.3% PBST for at least 1.5 hours, and incubated with primary antibodies diluted in 0.3% PBST at 4°C for 2 days. Samples were washed again 4 times for 15 minutes in 0.3% PBST at RT, and incubated with secondary antibodies for 2 days in darkness. Samples were then washed in 0.3% PBST for 4 times, balanced in SlowFade Gold antifade reagent (ThermoFisher, S36937) for 1 hour and mounted on a slide (ThermoFisher Superfrost Plus, 4951PLUS4) using SlowFade Gold antifade reagent.

Antibodies used in this study were: rabbit anti-GFP (ThermoFisher, A11122, 1:1,000), rat anti-HA (Roche, 11867423001, 1:100), mouse anti-Bruchpilot (DSHB, 1:20), donkey anti-mouse AF647 (Invitrogen, 1900251, 1:1,000), donkey anti-rabbit AF488 (Invitrogen, 1927937, 1:1,000), and goat anti-rat AF555 (ThermoFisher, A-21434, 1:1,000). Secondary antibodies were diluted in 50% glycerol. Images were taken with a 40X oil objective using a Zeiss LSM880 confocal microscope and stitched with ZEN software. ROI selection and fluorescence quantification were done with Fiji (ImageJ 1.52i, http://fiji.sc). The hemispheres ipsilateral and contralateral to the amputations, and the SEZ ipsilateral and contralateral to the amputations, were selected as ROIs based on nc82 staining, using the freehand selection tool in ImageJ. The signals from second-order neurons were normalized to the nc82 channel by dividing the mean red signal over the mean nc82 signal (normalized red signal), and the ipsi/contra ratio was calculated as the ratio between the normalized red signal on the ipsilateral side and the normalized signal on the contralateral side.

### Calcium imaging

Flies were raised at 18°C for 14 days, and then switched to 1 mM ATR-supplemented food for at least 1 day prior to imaging. Flies in the amputated groups were anaesthetized on ice and amputated using microdissection scissors. Tarsal segments 1 through 5 and the tibia, or wings, were bilaterally ablated at least 3 days prior to imaging. After 3 days, the axonal terminals of the primary sensory neurons were completely degenerated, as indicated by the lack of EYFP signal in the corresponding neuropils.

Flies were cold-anaesthetized and inserted into a triangular hole in an aluminum shim glued to a custom-made polycarbonate holder. The head and the body were separated by the aluminum shim, and the abdomen, thorax and the head were glued to the aluminum shim using UV curing glue (KOA300, Kemxert) to minimize motion. The head was covered with Adult Hemolymph-like saline buffer (in mM: 103 NaCl, 3 KCl, 5 HEPES, 8 Trehalose, 10 Glucose, 26 NaHCO_3_, 1 NaH_2_PO_4_, 1.5 CaCl_2_, 4 MgCl_2_), and the proboscis and the cuticle in the head were removed to expose the SEZ. The esophagus was also removed to expose the SEZ and to reduce motion.

Optogenetic stimulation was produced by a 617 nm LED (Thorlabs, M617F2), and delivered approximately 0.5 cm away from the thorax of the fly via a Ø2.5 mm (FC) Ferrule Patch Cable (Thorlabs, M126L01) and a Ø400 μm Core, 0.5NA fiber optic cannula (CFMC54L20). Light stimulation was 2 seconds long, controlled by a T-cube LED driver (Thorlabs, LEDD1B) and an Arduino Uno microcontroller. The LED was used at maximum intensity.

Images were acquired with a CCD camera (Retiga-R3, QImaging) mounted on a widefield fluorescence microscope (Olympus BX40) equipped with a 40x water immersion objective (Olympus LUMPLFLN 40XW, 0.8 NA). Excitation light was produced with a LED light (CoolLED pE-100). Image size was 960 x 730 pixels. Exposure time was 250 ms, acquired at 4 Hz. Micromanager 1.4 was used to control the camera, excitation light and data acquisition.

Videos were processed using NIH ImageJ. A 240-pixel diameter ROI was selected covering the SEZ (SEZ ROI). Another 40-pixel ROI was selected in a region lacking GFP signal (background ROI). ΔF/F was calculated as (F_t_ − F_0_)/F_0_, in which F_t_ is the mean fluorescence intensity in the SEZ ROI (after subtracting the mean fluorescence intensity in the background ROI) at time t, and F_0_ is the mean fluorescence intensity (after subtracting the same background intensity) during the 20 frames prior to optogenetic stimulation.

## Supporting information

S1 Fig Chemosensory gene expression in the wings of control and *Poxn* flies. (A) *IR* genes. (B) *Gr* genes. (C) *Or* genes. Genes are listed in decreasing order of FPKM in the control genotype *(w*^*1118*^ *Canton-S)*. Genes are shown only if the mean FPKM > 0.05 for the control samples. Error bars indicate SEM. Underlying data for this figure can be found in S2 Data. FPKM, fragments per million mapped reads per kilobase of gene length; Gr, gustatory receptor; IR, ionotropic receptor.
(TIF)

S2 Fig Chemosensory gene expression in the wings of control and *Poxn* flies. (A) *ppk* genes. (B) *Trp* genes (C) *CheB* genes, which are a class of *CSP* genes. (D) *CheA* genes, a class of *CSP* genes. (E) *Obp* genes. Genes are listed in decreasing order of FPKM in the control genotype *(w*^*1118*^ *Canton-S)*. Genes are shown only if the mean FPKM > 0.05 for the control samples. Error bars indicate SEM Underlying data for this figure can be found in S2 Data. CSP, chemosensory protein; FPKM, fragments per million mapped reads per kilobase of gene length; Obp, odorant binding protein; ppk, pickpocket; Trp, transient receptor potential.
(TIF)

S3 Fig qPCR of *IR52a* in the wing. qPCR of *IR52a* in male wings (blue) and female wings (red), using two set of primers. In the case of each primer pair, the transcription level was normalized to eIF1α, used as an internal control. The male value was then normalized to the female value. *n* = 3; the error bar indicates SEM. Underlying data for this figure can be found in S2 Data. IR, ionotropic receptor; qPCR, quantitative PCR.
(TIF)

S4 Fig Expression pattern of *IR52a-GAL4* in males and females. Expression of *IR52a > mCD8GFP* in the male (A) and female (B) wing, and in the male (C) and female (D) ventral nerve cord, showing leg (arrows) and wing (arrowheads) neuropils. GFP, green fluorescent protein; IR, ionotropic receptor.
(TIF)

S5 Fig Wing *IR52a^+^* neurons express *fruitless*, a marker of sexual circuitry. (A–C) Male wing labeled in green by *IR52a-GAL4*/*UAS-Stinger* (a nuclear GFP), and in red by *fru-LexA*/*LexAop-tomato-nls*. Arrows indicate neurons that clearly express both GFP and tdTomato. GFP, green fluorescent protein; IR, ionotropic receptor.
(TIF)

S6 Fig Dye labeling. (A) Fly prior to labeling. (B) Fly after spending 30 minutes in a chamber with Nile red. Fluorescence is most easily visible on the ventral abdomen.
(TIF)

S7 Fig The *IR52* gene cluster and the generation of an *IR52a* deletion. (A) *IR52* cluster contains five genes: *IR52a*, *IR52b*, *IR52e*, *IR52c*, and *IR52d*. The *D*. *melanogaster* reference genome contains only four genes in the *IR52* cluster [84]. Our Canton-S5 stock contains five genes: *IR52b* in the reference genome is an in-frame fusion of the *IR52b* and *IR52e* genes found in our stock [15]. (B) The *IR52a* CRISPR deletion. There are 599 amino acids in the predicted IR52a protein. The deletion removes codons specifying amino acids 31 to 447, which accounts for approximately 70% of the amino acid sequence. (C) Primers used in constructing deletion. Primers 1–2 were used for creating the CRISPR Guide chiRNA, and primers 3–10 were used for constructing the CRISPR donor plasmid. IR, ionotropic receptor.
(TIF)

S8 Fig *IR52a* shows normal locomotion behavior. Mean height (+/− SEM) reached by male flies (A) and female flies (B) in 5 seconds in a climbing assay. None of the lines varied significantly from the controls, ANOVA, *n* = 13–20 for males, *n* = 17–19 for females. (C) Mean +/− SEM speed of male flies during courtship behaviors. n.s., not significant, ANOVA, *n* = 26–34 each genotype. Underlying data for this figure can be found in S2 Data. IR, ionotropic receptor.
(TIF)

S9 Fig Disruption of *IR52a* in females did not affect wing extension behavior of males. ANOVA, *n* = 35–43. Underlying data for this figure can be found in S2 Data. IR, ionotropic receptor.
(TIF)

S10 Fig IR52a^+^ axons have degenerated three days after foreleg ablation. Expression of GFP in an *IR52a > mCD8GFP* fly three days after foreleg ablation. Notice the intact axons in the wing neuropil (arrow) and midleg neuropil (arrowhead), and the absence of GFP in the foreleg neuropil (indicated by the red oval). GFP, green fluorescent protein; IR, ionotropic receptor.
(TIF)

S11 Fig Wing IR52a^+^ neurons synapse with neurons in the SEZ. (A) Unilateral leg ablations in male flies engineered to allow *trans*-Tango labeling. After ablating legs on one side (the right side in this case), GFP signal (produced in IR52a^+^ neurons) is lost from the foreleg, midleg and hindleg neuropils on the right side, but it remains in the wing neuropil (see Fig 7A for map of neuropils). The mtdTomato signal (produced in neurons post-synaptic to IR52a^+^ neurons) in the SEZ is shown in the boxed area and at higher magnification below (yellow arrows). (B) Unilateral ablations of legs and wing. Note the lack of GFP signal in the foreleg, wing, midleg and hindleg neuropils. The mtdTomato signal in the SEZ ipsilateral to the ablations (right side) is lower than that on the contralateral (left) side (yellow arrows). (C) Quantification of the ratio of mtdTomato signal in the ipsilateral and contralateral hemispheres, following each kind of ablation. The ratio is lower when wing and legs are ablated, compared to the ratio when legs only are ablated. (*P* < 0.01; *t* test). (D) Quantification of the ratio of mtdTomato signal in the ipsilateral and contralateral SEZ, following each kind of ablation. The ratio is lower when wing and legs are ablated, compared to the ratio when legs only are ablated (*P* < 0.05). Underlying data for this figure can be found in S2 Data. GFP, green fluorescent protein; IR, ionotropic receptor; SEZ, subesophageal zone.
(TIF)

S1 Table Differentially expressed genes between control and *Poxn*. Columns C and D indicate FPKM. *P* values and adjusted *P* values are given by DESeq2 Wald test. FPKM, FPKM, fragments per million mapped reads per kilobase of gene length.
(XLSX)

S1 Data Quantitative observations that underlie the figures in main text. (XLSX)

S2 Data Quantitative observations that underlie the figures in supplementary information. (XLSX)

## Abbreviations

ATR: all-trans-retinal
CNS: central nervous system
CSP: chemosensory protein
Fru: fruitless
GO: gene ontology
Gr: gustatory receptor
IR: ionotropic receptor
mw: molecular weight
Obp: odorant binding protein
Ppk: pickpocket
qPCR: quantitative PCR
RT-PCR: reverse transcriptase PCR
SEZ: subesophageal zone
SLP: superior lateral protocerebrum
Trp: transient receptor potential
VLP: ventrolateral protocerebrum
VNC: ventral nerve cord
