# Supplementary figures and images for: Chemosensory sensilla of the Drosophila wing express a candidate ionotropic pheromone receptor
Source: PLoS Biol. 2019 May 21;17(5):e2006619. doi: 10.1371/journal.pbio.2006619 (PMC6528970; doi:10.1371/journal.pbio.2006619)

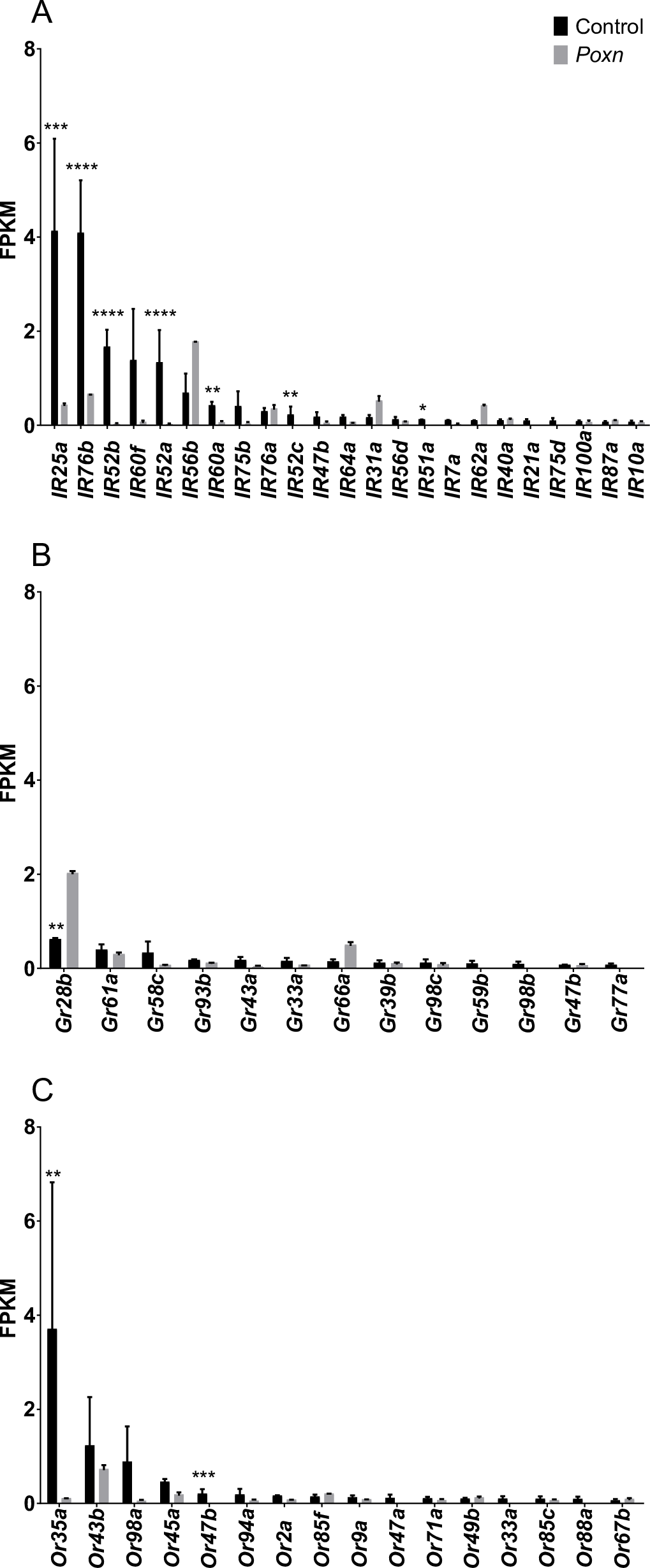

Supplement: S1 Fig — (A) IR genes. (B) Gr genes. (C) Or genes. Genes are listed in decreasing order of FPKM in the control genotype (w1118 Canton-S). Genes are shown only if the mean FPKM > 0.05 for the control samples. Error bars indicate SEM. Underlying data for this figure can be found in S2 Data. FPKM, fragments per million mapped reads per kilobase of gene length; Gr, gustatory receptor; IR, ionotropic receptor. (TIF) [file pbio.2006619.s001.tif]

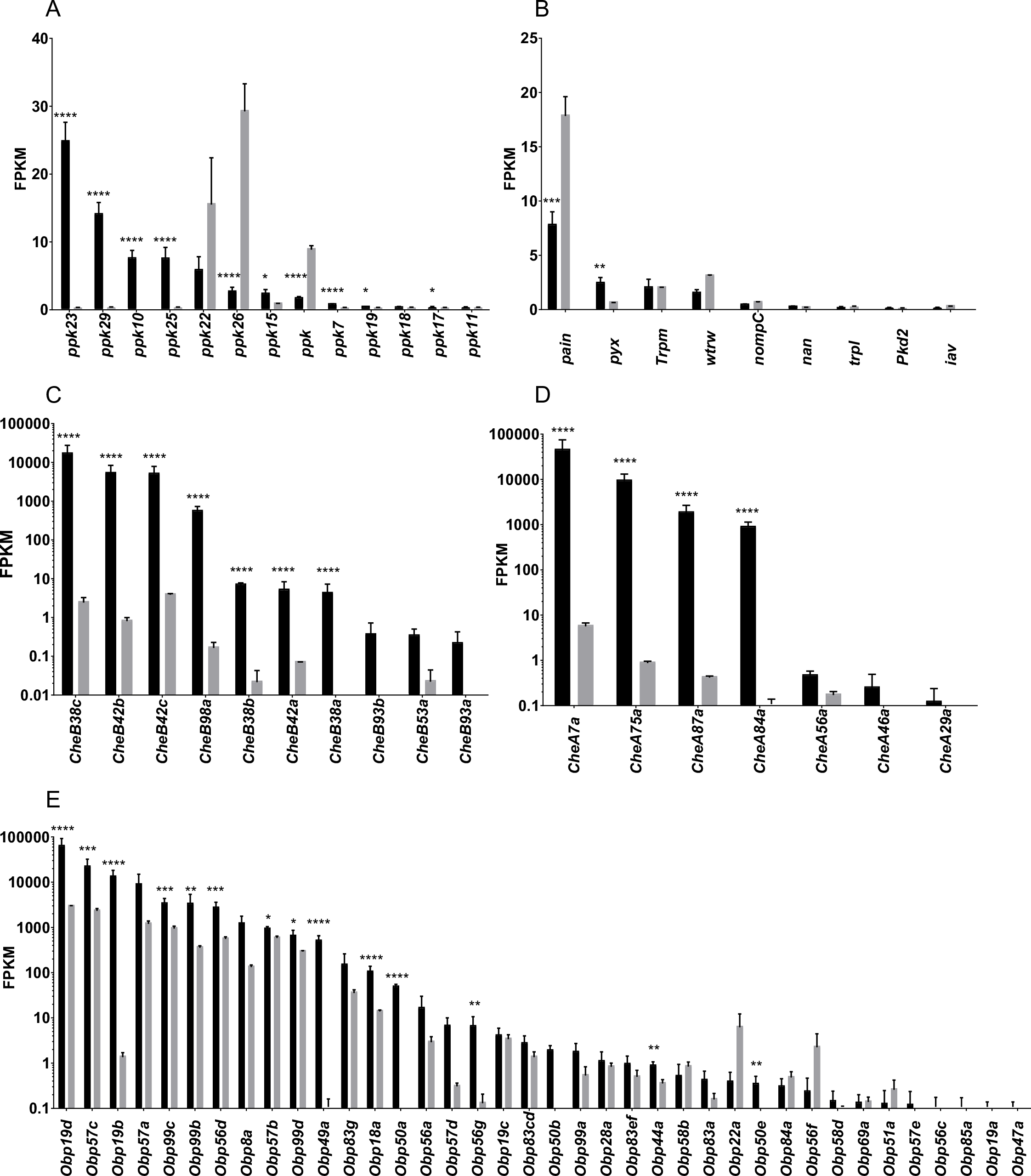

Supplement: S2 Fig — (A) ppk genes. (B) Trp genes (C) CheB genes, which are a class of CSP genes. (D) CheA genes, a class of CSP genes. (E) Obp genes. Genes are listed in decreasing order of FPKM in the control genotype (w1118 Canton-S). Genes are shown only if the mean FPKM > 0.05 for the control samples. Error bars indicate SEM Underlying data for this figure can be found in S2 Data. CSP, chemosensory protein; FPKM, fragments per million mapped reads per kilobase of gene length; Obp, odorant binding protein; ppk, pickpocket; Trp, transient receptor potential. (TIF) [file pbio.2006619.s002.tif]

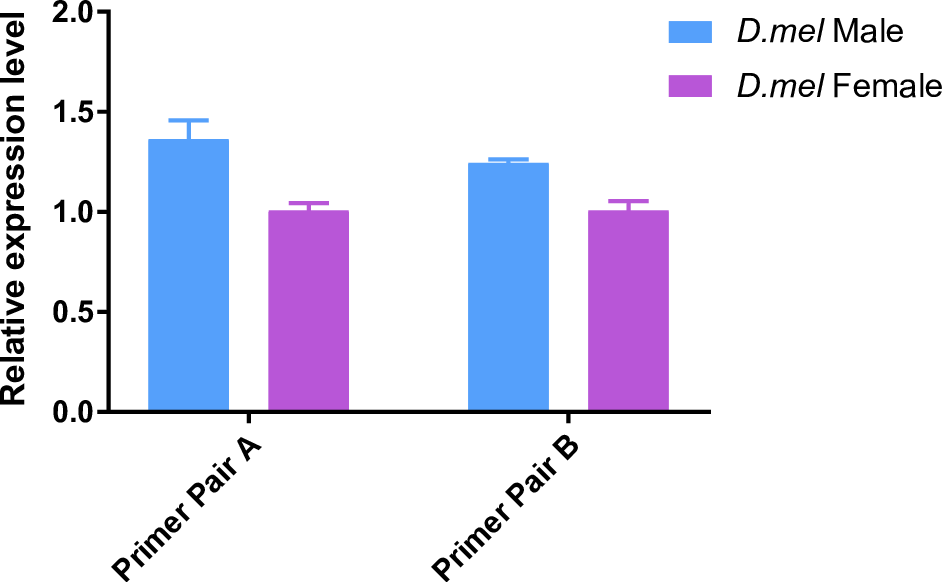

Supplement: S3 Fig — qPCR of IR52a in male wings (blue) and female wings (red), using two set of primers. In the case of each primer pair, the transcription level was normalized to eIF1α, used as an internal control. The male value was then normalized to the female value. n = 3; the error bar indicates SEM. Underlying data for this figure can be found in S2 Data. IR, ionotropic receptor; qPCR, quantitative PCR. (TIF) [file pbio.2006619.s003.tif]

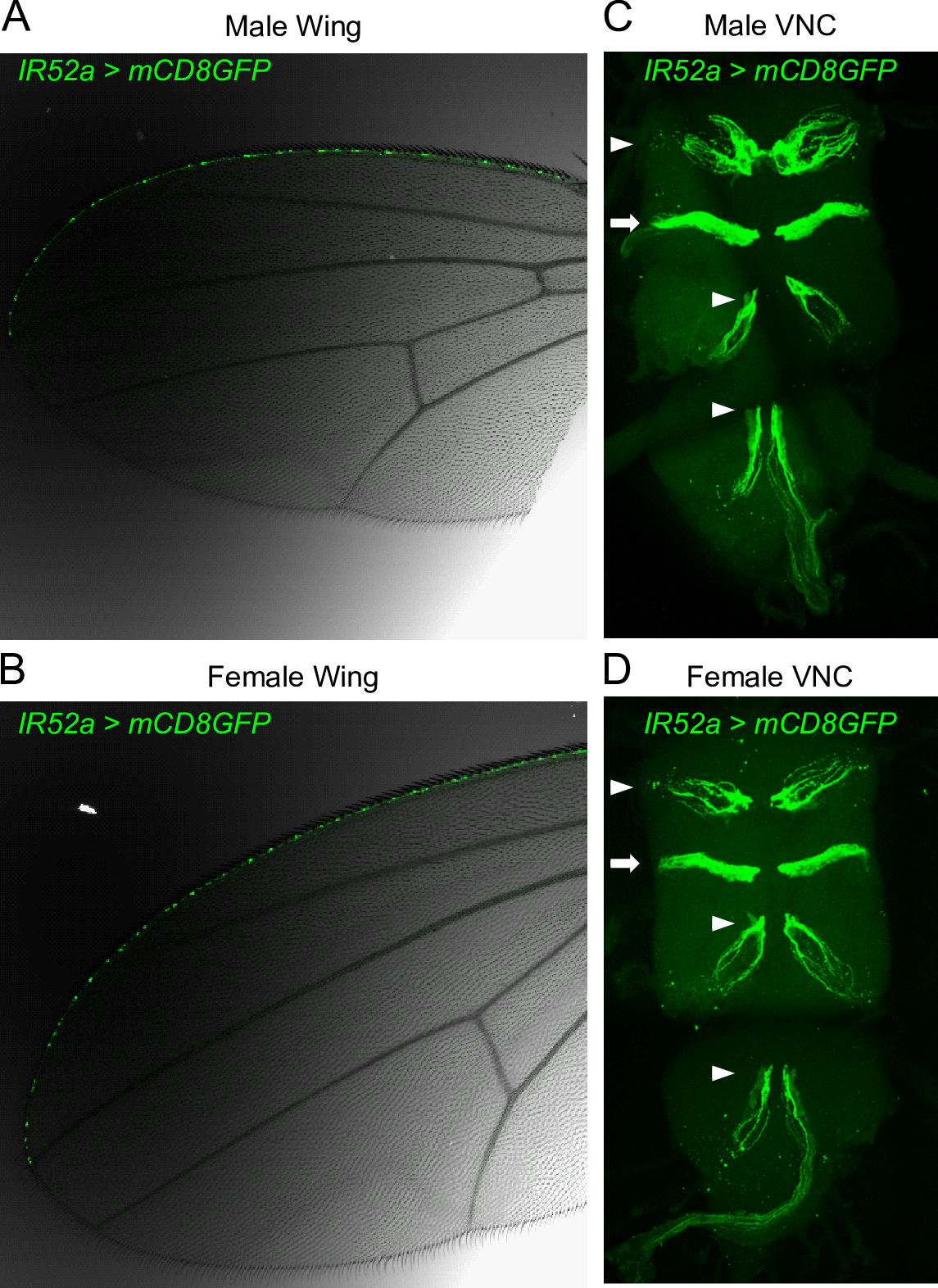

Supplement: S4 Fig — Expression of IR52a > mCD8GFP in the male (A) and female (B) wing, and in the male (C) and female (D) ventral nerve cord, showing leg (arrows) and wing (arrowheads) neuropils. GFP, green fluorescent protein; IR, ionotropic receptor. (TIF) [file pbio.2006619.s004.tif]

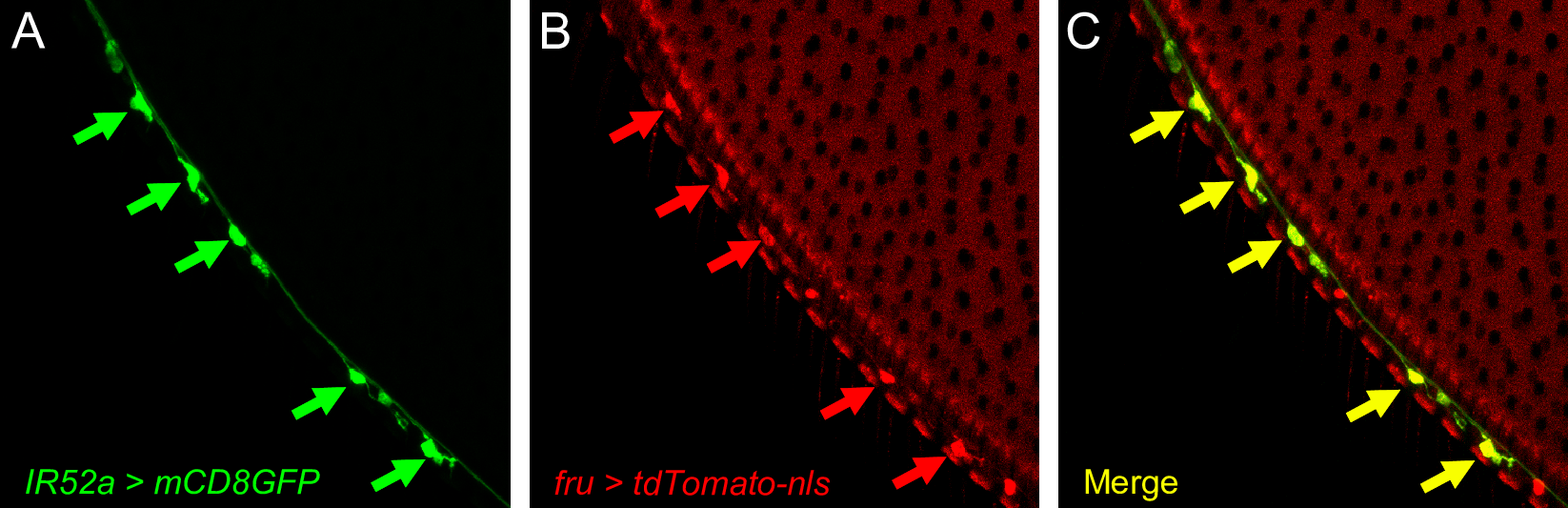

Supplement: S5 Fig — (A–C) Male wing labeled in green by IR52a-GAL4/UAS-Stinger (a nuclear GFP), and in red by fru-LexA/LexAop-tomato-nls. Arrows indicate neurons that clearly express both GFP and tdTomato. GFP, green fluorescent protein; IR, ionotropic receptor. (TIF) [file pbio.2006619.s005.tif]

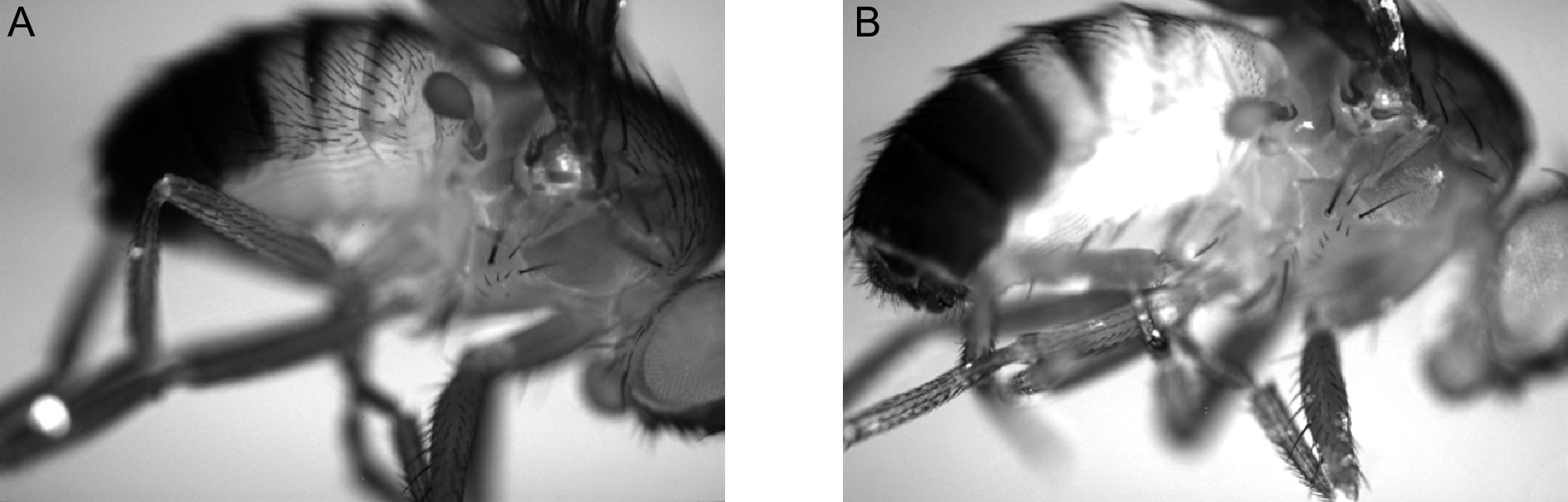

Supplement: S6 Fig — (A) Fly prior to labeling. (B) Fly after spending 30 minutes in a chamber with Nile red. Fluorescence is most easily visible on the ventral abdomen. (TIF) [file pbio.2006619.s006.tif]

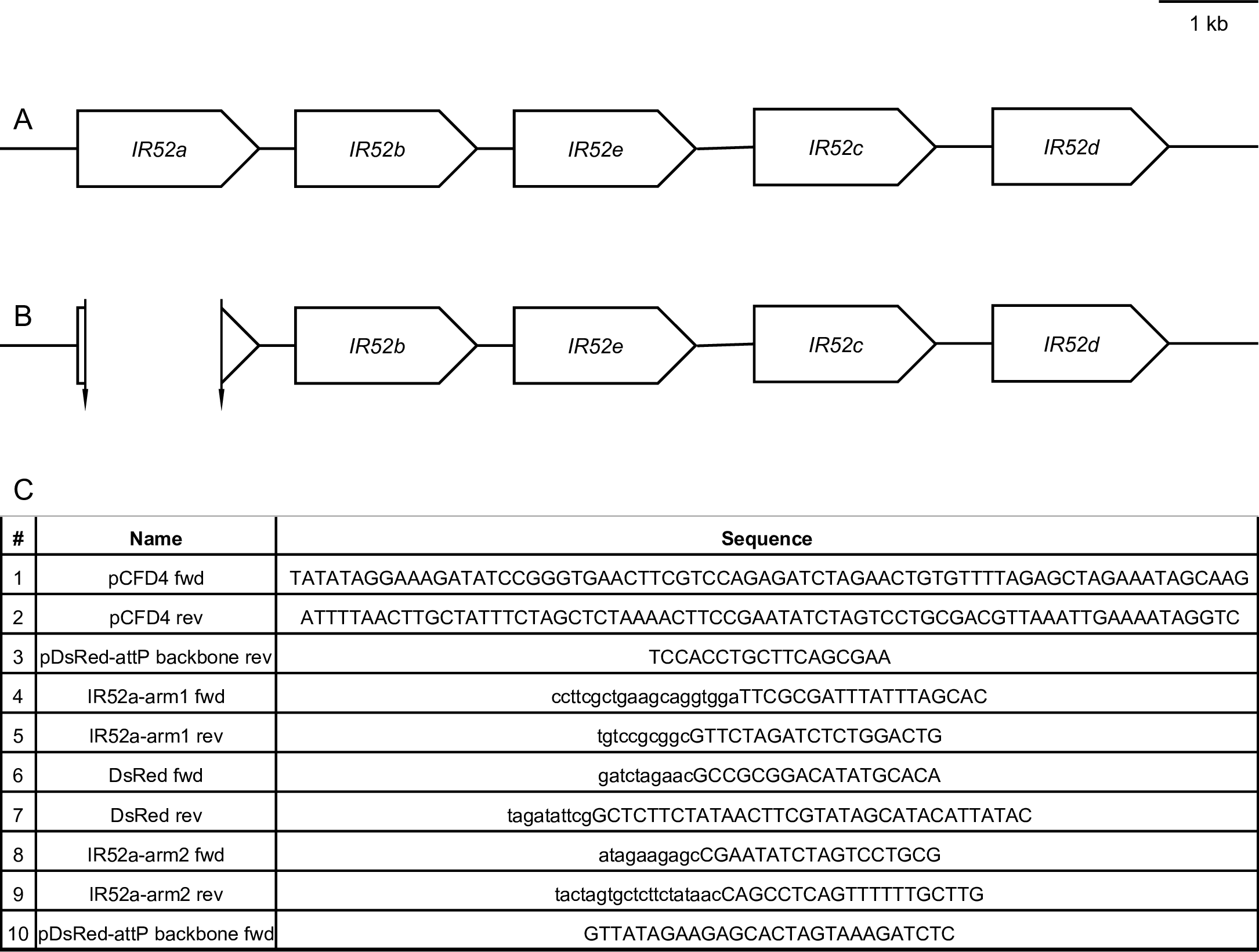

Supplement: S7 Fig — (A) IR52 cluster contains five genes: IR52a, IR52b, IR52e, IR52c, and IR52d. The D. melanogaster reference genome contains only four genes in the IR52 cluster [84]. Our Canton-S5 stock contains five genes: IR52b in the reference genome is an in-frame fusion of the IR52b and IR52e genes found in our stock [15]. (B) The IR52a CRISPR deletion. There are 599 amino acids in the predicted IR52a protein. The deletion removes codons specifying amino acids 31 to 447, which accounts for approximately 70% of the amino acid sequence. (C) Primers used in constructing deletion. Primers 1–2 were used for creating the CRISPR Guide chiRNA, and primers 3–10 were used for constructing the CRISPR donor plasmid. IR, ionotropic receptor. (TIF) [file pbio.2006619.s007.tif]

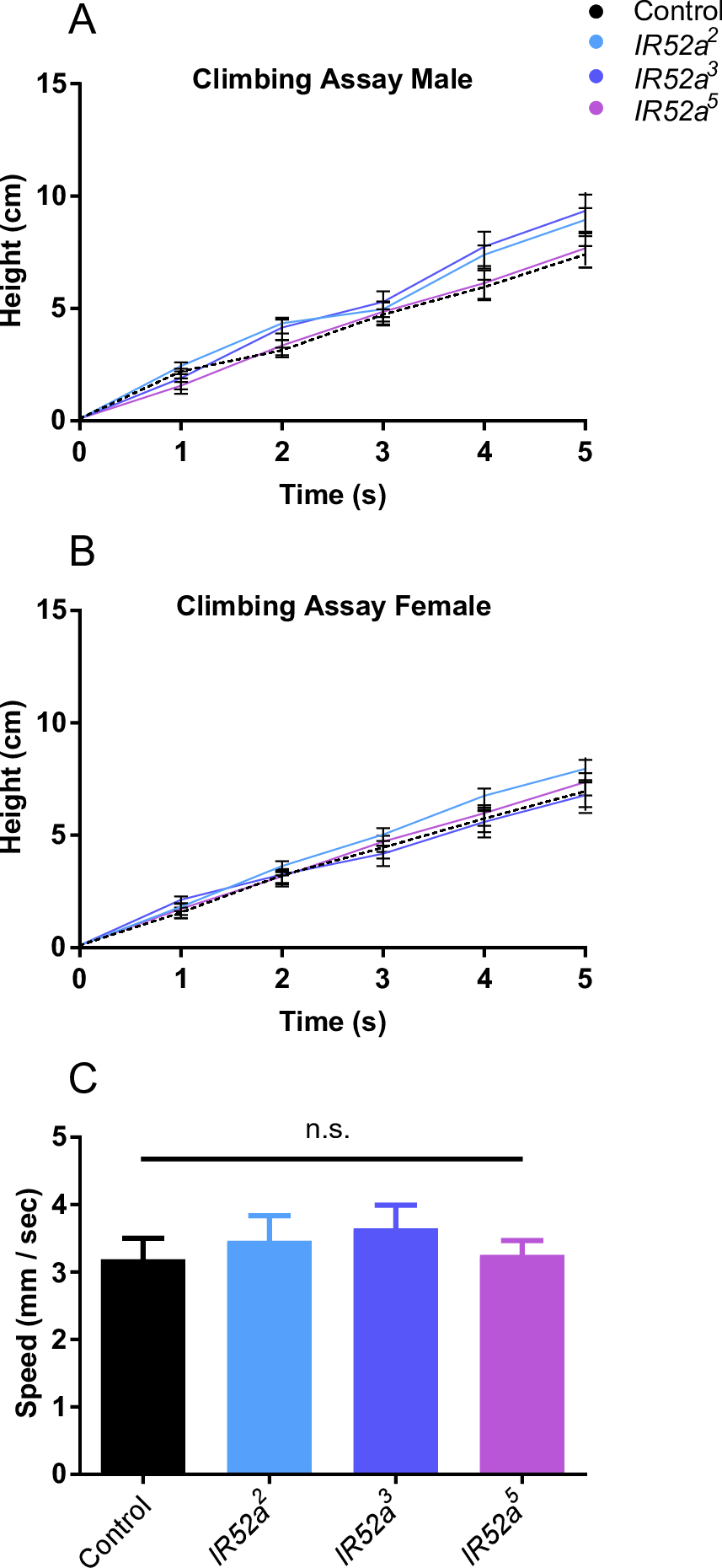

Supplement: S8 Fig — Mean height (+/− SEM) reached by male flies (A) and female flies (B) in 5 seconds in a climbing assay. None of the lines varied significantly from the controls, ANOVA, n = 13–20 for males, n = 17–19 for females. (C) Mean +/− SEM speed of male flies during courtship behaviors. n.s., not significant, ANOVA, n = 26–34 each genotype. Underlying data for this figure can be found in S2 Data. IR, ionotropic receptor. (TIF) [file pbio.2006619.s008.tif]

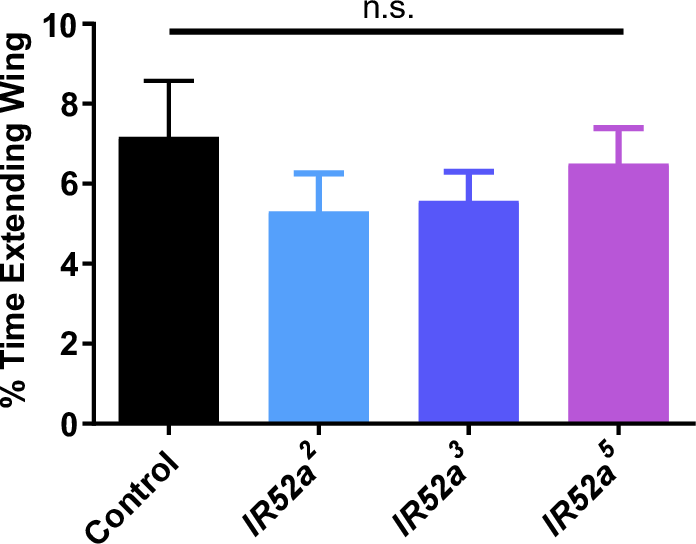

Supplement: S9 Fig — ANOVA, n = 35–43. Underlying data for this figure can be found in S2 Data. IR, ionotropic receptor. (TIF) [file pbio.2006619.s009.tif]

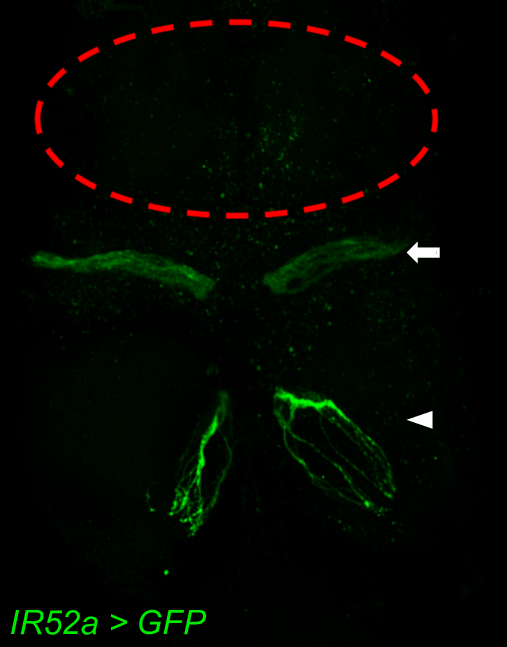

Supplement: S10 Fig — Expression of GFP in an IR52a > mCD8GFP fly three days after foreleg ablation. Notice the intact axons in the wing neuropil (arrow) and midleg neuropil (arrowhead), and the absence of GFP in the foreleg neuropil (indicated by the red oval). GFP, green fluorescent protein; IR, ionotropic receptor. (TIF) [file pbio.2006619.s010.tif]

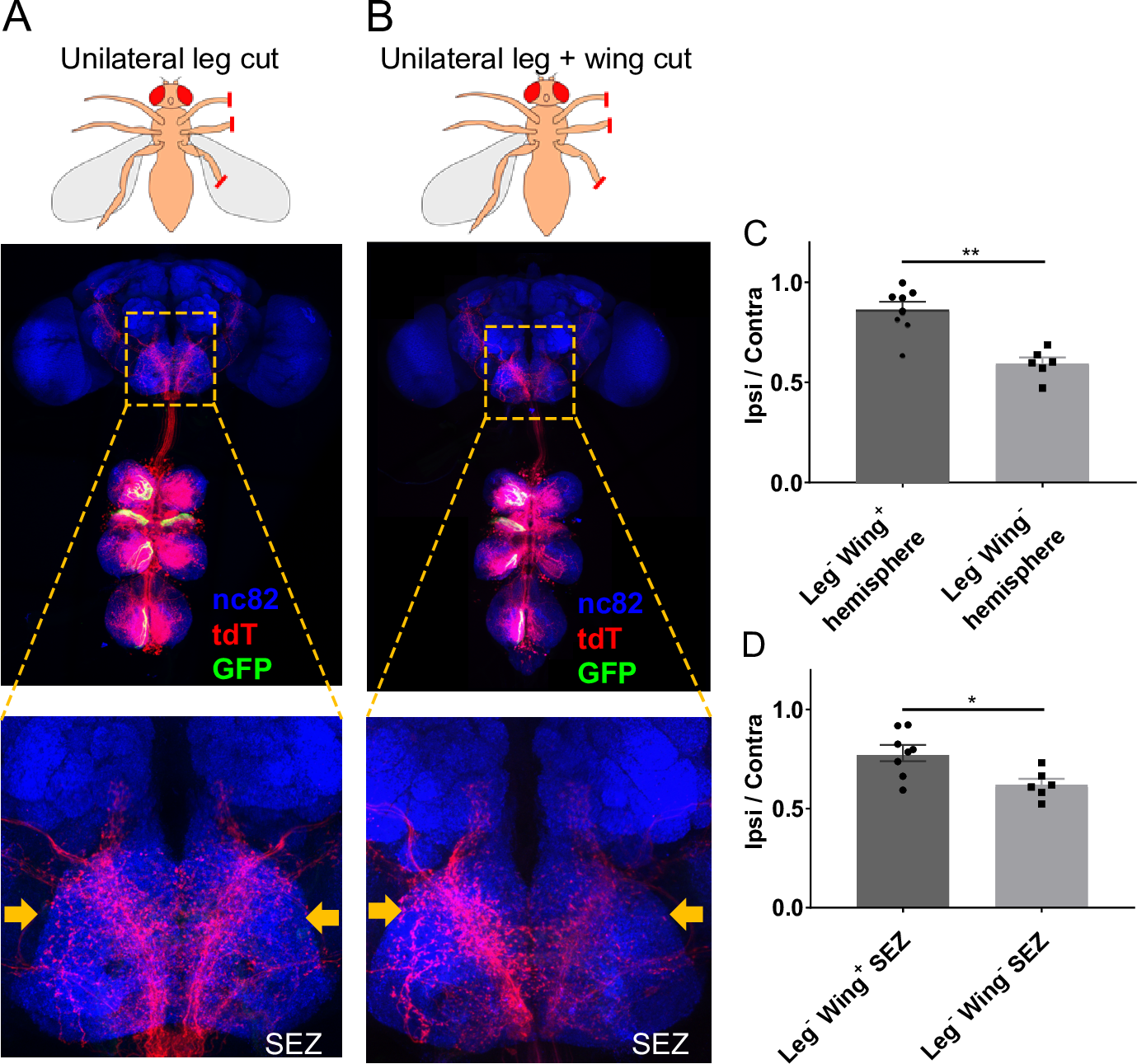

Supplement: S11 Fig — (A) Unilateral leg ablations in male flies engineered to allow trans-Tango labeling. After ablating legs on one side (the right side in this case), GFP signal (produced in IR52a+ neurons) is lost from the foreleg, midleg and hindleg neuropils on the right side, but it remains in the wing neuropil (see Fig 7A for map of neuropils). The mtdTomato signal (produced in neurons post-synaptic to IR52a+ neurons) in the SEZ is shown in the boxed area and at higher magnification below (yellow arrows). (B) Unilateral ablations of legs and wing. Note the lack of GFP signal in the foreleg, wing, midleg and hindleg neuropils. The mtdTomato signal in the SEZ ipsilateral to the ablations (right side) is lower than that on the contralateral (left) side (yellow arrows). (C) Quantification of the ratio of mtdTomato signal in the ipsilateral and contralateral hemispheres, following each kind of ablation. The ratio is lower when wing and legs are ablated, compared to the ratio when legs only are ablated. (P < 0.01; t test). (D) Quantification of the ratio of mtdTomato signal in the ipsilateral and contralateral SEZ, following each kind of ablation. The ratio is lower when wing and legs are ablated, compared to the ratio when legs only are ablated (P < 0.05). Underlying data for this figure can be found in S2 Data. GFP, green fluorescent protein; IR, ionotropic receptor; SEZ, subesophageal zone. (TIF) [file pbio.2006619.s011.tif]
